# Brazilian growth charts for 22q11.2 deletion syndrome from birth to 17 years

**DOI:** 10.1016/j.jped.2026.101560

**Published:** 2026-05-30

**Authors:** Amanda de Sousa Lima Strafacci, Fabio Bertapelli, Bruna Fagundes Rodrigues Alaite, Júlia Lôndero Heleno, Débora Gusmão Melo, Maria Isabel Riesemberg, Carolina de Souza Araújo, Fernanda Araújo Romera, Ana Carolina Xavier, Stela Carpini Dantas, Gil Guerra-Junior, Vera Lúcia Gil-da-Silva-Lopes, SD22q11.2 Research Group, Agnes Cristina Fett-Conte, Josiane de Souza, Luissa Hikari Hayashi Araujo, Maria Angelica de Faria Domingues de Lima, Paula Frassinetti Vasconcelos de Medeiros, Raquel Boy, Rayana Elias Maia, Têmis Maria Félix

**Affiliations:** h Faculdade de Medicina de São José do Rio Preto (FAMERP), Hospital de Base, Departamento de Biologia Molecular, São José do Rio Preto, SP, Brazil; i Pontifícia Universidade Católica do Paraná, Hospital Pequeno Príncipe, Curitiba, PR, Brazil; j Hospital do Trabalhador, Centro de Atendimento Integral ao Fissurado Labiopalatal, Curitiba, PR, Brazil; k Hospital Universitário Gaffrée e Guinle, Serviço de Genética Médica, Rio de Janeiro, RJ, Brazil; l Hospital Universitário Alcides Carneiro, Centro de Ciências Biológicas e da Saúde, Unidade Acadêmica de Medicina, Campina Grande, PB, Brazil; m Universidade Estadual do Rio de Janeiro, Departamento de Pediatria, Rio de Janeiro, RJ, Brazil; n Universidade Federal da Paraíba, Departamento de Pediatria e Genética, João Pessoa, PB, Brazil; o Hospital de Clínicas de Porto Alegre (HCPA), Serviço de Genética Médica, Porto Alegre, RS, Brazil; a Universidade Estadual de Campinas (UNICAMP), Faculdade de Ciências Médicas (FCM), Departamento de Pediatria, Campinas, SP, Brazil; b Universidade Federal de São Paulo (UNIFESP), Departamento de Ciências do Movimento Humano, Santos, SP, Brazil; c Universidade Estadual de Campinas (UNICAMP), Faculdade de Ciências Médicas (FCM), Departamento de Medicina Translacional, Campinas, SP, Brazil; d Universidade Federal de São Paulo (UNIFESP), Escola Paulista de Medicina, Departamento de Morfologia e Genética, Divisão de Genética, São Paulo, SP, Brazil; e Centro de Tratamento e Reabilitação de Fissuras Labiopalatinas, Centrinho Prefeito Luiz Gomes, Joinville, SC, Brazil; f Universidade Estadual de Campinas (UNICAMP), Faculdade de Ciências Médicas (FCM), Centro de Pesquisa em Pediatria (CIPED), Laboratório de Crescimento e Desenvolvimento (LabCreD), Campinas, SP, Brazil; g Universidade Estadual de Campinas (UNICAMP), Departamento de Genética Médica e Medicina Genômica, Campinas, SP, Brazil

**Keywords:** 22q11.2 deletion syndrome, DiGeorge syndrome, Growth charts, Body weight, Body height, Body mass index

## Abstract

**Objective:**

To develop Brazilian growth charts for weight, height, body mass index (BMI), and head circumference (HC), stratified by sex, for patients with 22q11.2 deletion syndrome (22q11.2DS) from birth to 17 years.

**Methods:**

This multicenter, retrospective, longitudinal study included 1826 anthropometric data collected through medical record review of 113 patients with a laboratory-confirmed diagnosis of 22q11.2DS who were followed at 11 centers participating in the Craniofacial Brazil Project. The 3rd, 10th, 25th, 50th, 75th, 90th, and 97th percentiles for anthropometric parameters were developed for males and females from birth to 17 years old using the Lambda–Mu–Sigma (LMS) method and graphically compared with reference percentiles from the Centers for Disease Control and Prevention using the Tidyverse package in R software.

**Results:**

A growth pattern distinct from that of the reference population was observed, with more pronounced deficits in height and weight during the first two years of life in both sexes. After this period, partial recovery in height was observed, more evident in females, whereas the deficit remained more pronounced in males. Differences in BMI and HC were less marked.

**Conclusion:**

The Brazilian growth charts for 22q11.2DS provide more appropriate parameters for clinical monitoring and individualized care of this population.

## Introduction

22q11.2 deletion syndrome (22q11.2DS), formerly known as DiGeorge syndrome or velocardiofacial syndrome, is one of the most common chromosomal microdeletions, with an estimated prevalence ranging from 1 in 2000 to 1 in 4000 live births [1,2]. The syndrome results from a deletion in the 22q11.2 region, which contains approximately 40 functional genes and typically spans 1.5 to 3 Mb [1]. Most cases arise from *de novo* events, although vertical transmission has also been reported [[1], [2], [3]]. The most common clinical manifestations include congenital heart disease, thymic and parathyroid hypoplasia, hypocalcemia, immunodeficiency, palatal abnormalities, marked feeding difficulties in early life, learning disabilities, and psychiatric disorders at different stages of life [[1], [2], [3], [4], [5], [6]].

With regard to growth, studies indicate that individuals with 22q11.2DS present more pronounced deficits in weight and height during early childhood, with a tendency toward partial recovery of final height when compared with the reference population [3,[5], [6], [7], [8], [9]].

Monitoring child growth is one of the cornerstone tools of health surveillance in pediatrics [10]. Growth charts proposed by the World Health Organization (WHO)[11] and the Centers for Disease Control and Prevention (CDC)[12] are the most widely used for this purpose, given their simplicity, low cost, and high accuracy [11,12]. However, these charts are based on reference populations and do not account for the specific growth patterns associated with genetic syndromes, which may lead to misinterpretation of weight and height deficits or excesses in these conditions [6,[11], [12], [13]].

In this context, several authors have advocated the development of syndrome-specific growth charts to provide parameters that more accurately reflect the clinical reality of each genetic condition [[6], [7], [8],[13], [14], [15], [16]]. To date, however, no growth charts based on data from Brazilian patients with 22q11.2DS have been published, limiting the availability of references adjusted to the anthropometric and ethnic characteristics of this population.

Therefore, the present study aimed to develop sex-specific growth charts for weight, height, body mass index (BMI), and head circumference (HC) for the Brazilian population with 22q11.2DS.

## Case series and methods

The study was conducted in accordance with the ethical principles of the Declaration of Helsinki and Brazilian National Health Council Resolution No 466/12 and was approved by the Research Ethics Committee for Human Beings of the State University of Campinas (UNICAMP) (CAAE: 35,316,314.9.1001.5404 and 85,020,018.8.0000.5404), as well as by the participating centers. Written informed consent was obtained from all participants or their legal guardians.

This was a multicenter, retrospective, longitudinal study. Data were collected through review of medical records from patients followed within the Craniofacial Brazil Project, which includes individuals receiving care at 11 centers located in the South, Southeast, and Northeast regions of Brazil. Due to the rarity of the condition, no time restriction was applied, and all patients meeting the inclusion criteria were initially included, resulting in anthropometric data spanning from 1991 to 2025.

Given the retrospective nature of the study, the database was constructed in two stages. In the first stage, all eligible patients had their anthropometric data manually transcribed in chronological order from medical records into a standardized data collection form developed in an Excel spreadsheet, which included safeguards to reduce manual entry errors. In addition to anthropometric measurements, relevant clinical data were also recorded for each patient. In the second stage, all collected data were unified and systematically reviewed by one author (ASLS). Individual data points were then assessed for the application of exclusion criteria 1 through 6. Exclusion criterion 7 was applied based on preliminary statistical analysis, which identified anthropometric values exceeding ±5 standard deviations relative to age and sex distribution. This two-step approach was adopted to minimize the inclusion of data affected by recording errors or incomplete information.

For standardization and database construction, the following variables were collected: (1) sex (male or female); (2) date of birth and date of clinical visit, allowing calculation of age; (3) weight (kg); (4) height (cm); and (5) head circumference (HC, cm). Body mass index (BMI, kg/m²) was calculated as weight (kg)/height² (m²). The standardized data collection form also included fields for diagnostic methods used to confirm 22q11.2DS, history of prematurity, birth data, hypothyroidism, growth hormone deficiency, congenital heart disease, scoliosis, hypoparathyroidism, and other comorbid conditions.

Inclusion criteria were patients aged from birth to 20 years with a diagnosis of 22q11.2DS confirmed by at least one of the following methods recognized as standard for microdeletion diagnosis: fluorescence in situ hybridization (FISH), multiplex ligation-dependent probe amplification (MLPA), chromosomal microarray analysis (CMA), exome sequencing, noninvasive prenatal testing (NIPT), or genome sequencing [17,18].

Exclusion criteria were as follows: (1) data in which age or date of evaluation could not be determined; (2) data from children aged 0 to 2 years with a history of prematurity or with unavailable information regarding prematurity; (3) data from individuals with untreated hypothyroidism; (4) data from individuals with untreated growth hormone (GH) deficiency; (5) data from individuals with congenital heart disease requiring treatment who had not been treated; (6) duplicate measurements from the same patient obtained within an interval of <1 month for ages 0 to 23 months or <12 months for ages 2 to 20 years; and (7) anthropometric measurements exceeding ±5 standard deviations relative to age and sex.

The LMS (Lambda Mu Sigma) method was used to model the curves [19]. Percentiles 3, 10, 25, 50, 75, 90, and 97 were generated for the construction of weight-for-age, height-for-age, WC-for-age, and BMI-for-age curves. The parameters L, M, and S correspond to the Box–Cox transformation power, the median, and the coefficient of variation, respectively. The LMS method accounts for skewness by assuming a Box–Cox normal distribution. Model selection was guided by goodness-of-fit criteria and by identifying the most appropriate combination of equivalent degrees of freedom (edf) for cubic spline smoothing. Age was entered using the ‘age original’ setting, without transformation or rescaling. Initial edf values were set to L = 3, M = 5, and S = 3. Model refinement followed LMSchartmaker Pro recommendations [20]. Final L, M, and S estimates, along with smoothed percentile curves, were exported from LMSchartmaker to spreadsheet software for the construction of tables and graphical outputs.

For graphical comparison between the growth charts for 22q11.2DS developed in this study and those of a reference population, CDC data from birth to 17 years[12] were used and implemented using the Tidyverse package[21] in R software, version 4.4.1 [22]. The CDC growth reference was selected because it provides a continuous reference across the full pediatric age range included in this study and is based on a large population of children without specific clinical conditions affecting growth. In addition, there are currently no Brazilian LMS-based reference curves covering the entire age range analyzed, which precluded comparison with a Brazilian reference population using the same methodological framework.

## Results

Between May 2024 and January 2025, 121 medical records of patients with 22q11.2DS were reviewed across 11 participating centers. Of these, 119 patients were initially included, yielding 3149 growth measurements (1353 wt, 1229 height, and 567 HC measurements) from birth to 20 years; 70 patients were female, and 49 were male. At this stage, all clinical variables relevant to the study were collected. Subsequently, exclusion criteria 1 through 6 were applied, followed by application of exclusion criterion 7 after preliminary statistical analysis. Insufficient data were available in the 18–20-year age range to allow reliable chart construction, and these data were therefore excluded. Overall, 1323 measurements (613 wt, 532 height, and 178 HC measurements) and six patients were excluded. Exclusion criterion 6 accounted for the largest proportion of excluded data (936 measurements), followed by criteria 2 (187 measurements) and 7 (166 measurements). No exclusions were attributable to criteria 3, 4, or 5. These data are detailed in Supplementary Table 1.

A total of 1826 growth measurements from 113 patients remained in the final analysis, including 66 females with 1074 measurements (447 wt, 407 height, and 220 HC measurements) and 47 males with 752 measurements (293 wt, 290 height, and 169 HC measurements). Based on the corresponding weight and height data, 644 BMI values were calculated (379 for females and 265 for males). Descriptive statistics, including the number of measurements, minimum, maximum, mean, and standard deviation, stratified by anthropometric parameters (weight, height, BMI, and HC), sex, and age group (birth to 24 months and 2 to 17 years), are presented in Supplementary Tables 2–7.

Sex-specific growth charts were constructed for weight-for-age, height-for-age, and head circumference–for-age from birth to 24 months, and for weight-for-age, height-for-age, and BMI-for-age from 2 to 17 years, in accordance with the proposed statistical modeling. These charts are presented in Supplementary Fig.s 1–12 and in Supplementary Tables 8–19, which report the corresponding LMS values and the 3rd, 10th, 25th, 50th, 75th, 90th, and 97th percentiles used to construct the charts.

**Figure 1:**
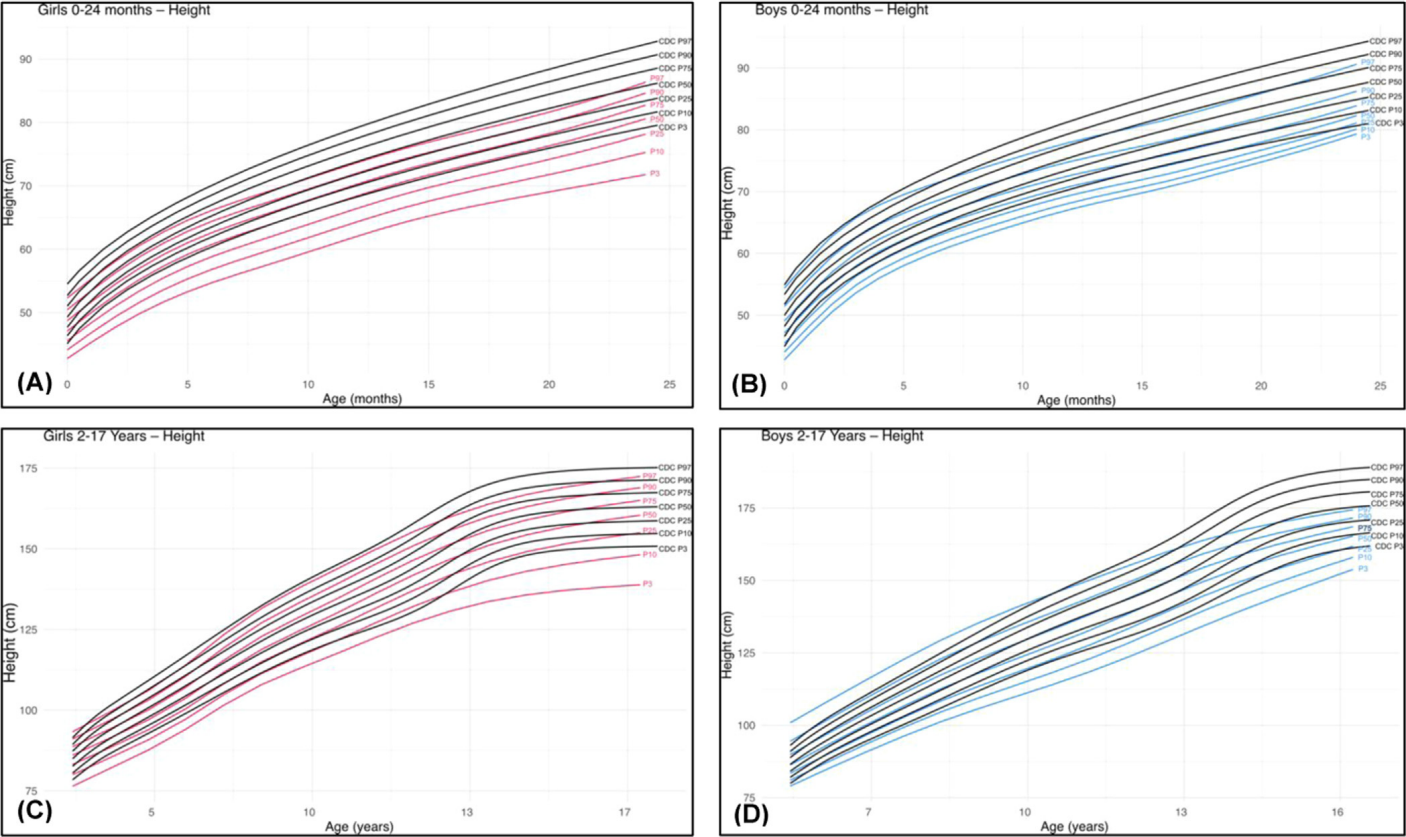
Comparative height charts for individuals with 22q11.2DS and CDC reference data (black) [12]: (A) females (red) from birth to 24 months; (B) males (blue) from birth to 24 months; (C) females (red) from 2 to 17 years; (D) males (blue) from 2 to 17 years.

**Figure 2:**
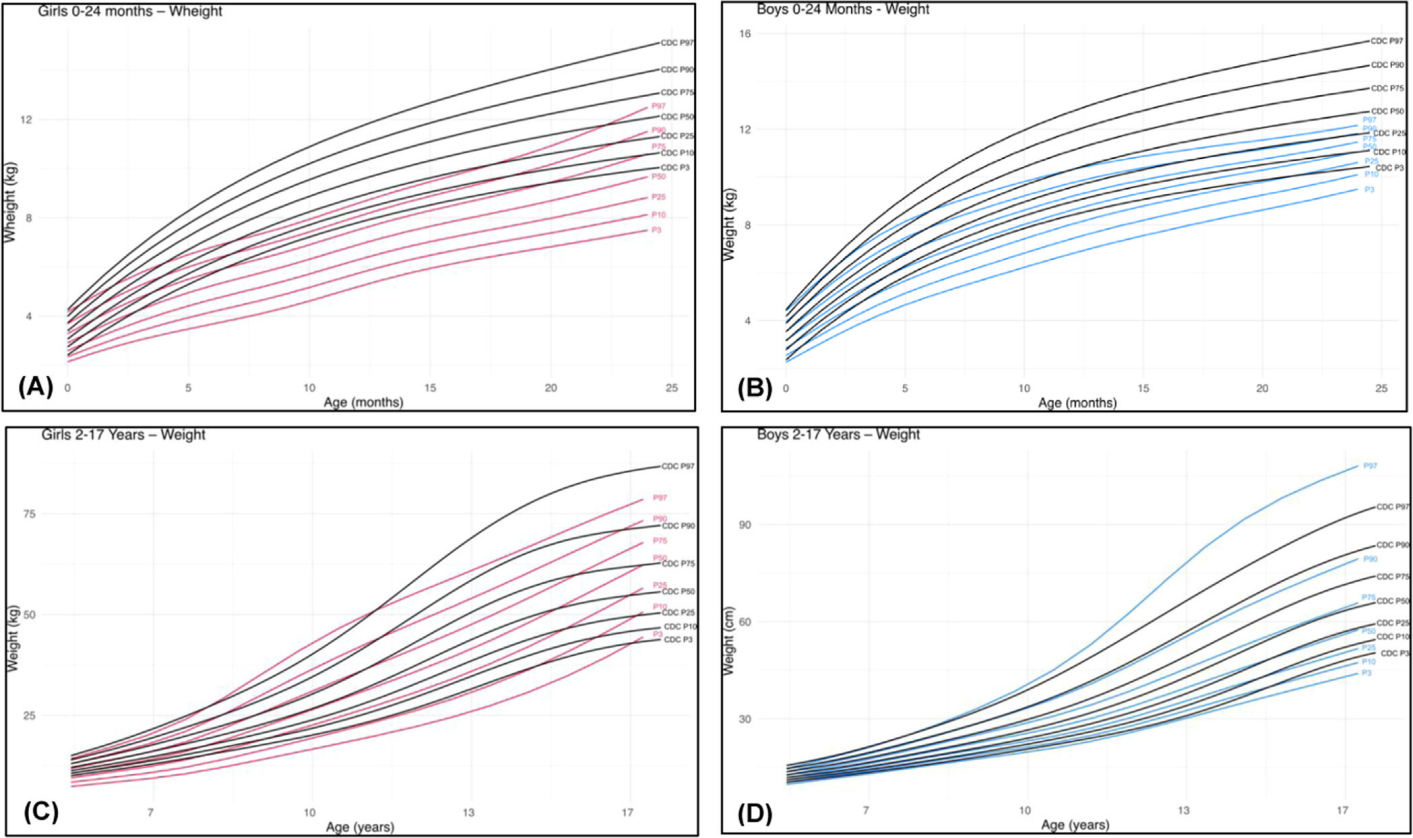
Comparative weight charts for individuals with 22q11.2DS (green) and CDC reference data (black) [12]: (A) females (red) from birth to 24 months; (B) males (blue) from birth to 24 months; (C) females (red) from 2 to 17 years; (D) males (blue) from 2 to 17 years.

**Figure 3:**
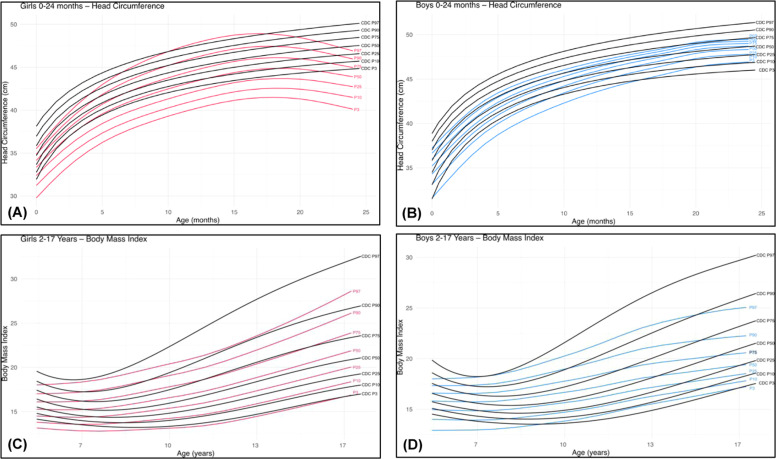
Comparative charts for individuals with 22q11.2DS and CDC reference data (black):[12] (A) head circumference, females (red) from birth to 24 months; (B) head circumference, males (blue) from birth to 24 months; (C) BMI (kg/m^2^), females (red) from 2 to 17 years; (D) BMI (kg/m^2^), males (blue) from 2 to 17 years.

Graphical comparisons between the sex-specific height charts developed in this study and the Centers for Disease Control and Prevention (CDC) reference charts[12] for healthy children and adolescents are shown in Figure 1–3 of the main article.

From birth to 24 months, clear differences were observed between the growth charts developed in this study and the CDC reference charts [12]. For height (Figure 1A and 1B), in both sexes, the 50th percentile of the 22q11.2DS population corresponded approximately to the 3rd percentile of the reference population. At 24 months of age, the median height for females with 22q11.2DS was 80.6 cm compared with 86.2 cm in the reference population [12], and for males, 82.3 cm versus 87.7 cm, respectively, representing a difference of nearly 6 cm in median height for both sexes (Supplementary Table 2).

For weight (Figure 2A and 2B), similar but more pronounced differences were observed, particularly among females. The 50th percentile of weight in females with 22q11.2DS fell below the 3rd percentile of the reference population (Figure 2A), whereas in males, the 50th percentile corresponded approximately to the 3rd percentile of the reference (Figure 2B). At 24 months, the median weight for females with 22q11.2DS was 9.7 kg compared with 12.1 kg in the reference population [12], and for males, 11.0 kg versus 12.7 kg [12], corresponding to differences of 2.4 kg in females and 1.7 kg in males (Supplementary Table 3).

Differences in HC were less pronounced (Figure 3A and 3B). In females, the 50th percentile lay between the 3rd and 10th percentiles of the reference population, whereas in males, the 50th percentile was similar in both populations. Notably, the 97th percentile of HC in the 22q11.2DS population corresponded to the 75th percentile of the reference population (Figure 3B).

From 2 to 17 years, differences between the growth charts developed in this study and the CDC reference charts[12] persisted. For height (Figure 1C and 1D), disparities were more pronounced in males. In males, the 50th percentile of the 22q11.2DS population corresponded approximately to the 10th percentile of the reference population (Figure 1D), whereas in females it corresponded to the 25th percentile (Figure 1C). At 17 years, the median height for males with 22q11.2DS was 165.3 cm compared with 175.3 cm in the reference population [12], representing a difference of 10 cm. In females, the median height was 160.4 cm in the 22q11.2DS group and 162.9 cm in the reference population [12], a difference of 2.5 cm (Supplementary Table 5).

For weight (Figure 2C and 2D), differences were again more pronounced in males, with the 50th percentile of the 22q11.2DS population corresponding to the 25th percentile of the reference population (Figure 2D). A similar pattern was observed for BMI (Figure 3C and 3D) in both sexes (Supplementary Table 7).

## Discussion

This study presents the first Brazilian growth charts for weight, height, HC, and BMI, stratified by sex and age from birth to 17 years, for individuals with 22q11.2DS. The differences observed in percentile correspondence indicate a distinct growth profile, characterized by growth deficits relative to the reference population [12]. Median height across all age groups and in both sexes was consistently lower than that of the reference population [12], with deficits being more pronounced during the first two years of life in both sexes and persisting in males after two years.

An additional aspect that warrants consideration is whether final adult height is achieved by 17 years of age in individuals with 22q11.2DS. Although the present analysis includes data up to this age, the retrospective design and lack of longitudinal follow-up to confirm growth cessation preclude definitive conclusions. Furthermore, the observed sex differences in growth trajectory, with more pronounced early impairment and subsequent catch-up in females compared to males, suggest potential differences in growth timing and maturation that merit further investigation in longitudinal studies.

The marked growth impairment observed in early childhood, followed by a tendency toward partial recovery at later ages, with an estimated prevalence of short stature of approximately 20%, is consistent with previously published data [3,6]. Lasprilla-Tovar et al [6]. reported a prevalence of short stature of 56.2% in individuals with 22q11.2DS when assessed using WHO reference charts; however, this prevalence decreased to 16.2% when 22q11.2DS-specific growth charts were applied, underscoring the importance of using tailored growth references for this population. Notably, the lack of height recovery in males after two years observed in the present study has not been previously described. Sex-specific differences in weight trajectories were also identified. Females showed more pronounced weight deficits during early childhood, followed by greater recovery at later ages, whereas males exhibited a more stable pattern across age groups. Although this pattern may suggest a relationship between early nutritional status and subsequent growth dynamics, including height trajectory, the present study does not allow for causal inferences. It is plausible that early feeding difficulties, which are common in individuals with 22q11.2DS, may differentially affect growth patterns between sexes. These findings reinforce the importance of considering sex-specific trajectories in the clinical assessment of these patients.

A similar sex-specific pattern was observed for HC. Females appeared to reach a plateau earlier than expected, whereas males followed a trajectory closer to the reference population. This finding should be interpreted with caution, as it may reflect methodological limitations, including reduced sample size in certain age groups. Nevertheless, the consistency of this pattern supports its reporting, and further longitudinal studies are needed to clarify its significance.

The mechanisms underlying this growth pattern remain incompletely understood. With respect to growth impairment, several authors suggest an interplay between intrinsic effects of the chromosomal deletion and systemic complications associated with the syndrome, acting independently or synergistically [2]. Feeding difficulties, which are typically more pronounced in early childhood, are thought to negatively influence growth and have been associated with hypotonia, cleft lip and palate, airway compromise, gastrointestinal dysfunction such as intestinal dysmotility, gastroesophageal reflux, dysphagia, and chronic constipation, as well as chronic illness and specific behavioral characteristics [1,3,[5], [6], [7],^9^].

Congenital heart disease does not appear to significantly interfere with growth [2]. Similarly, the impact of immunodeficiency on growth remains unclear. Ryan et al [5]. found no significant differences in growth between individuals with 22q11.2DS with and without immunodeficiency. The most frequently reported endocrine abnormalities include hypocalcemia and/or hypoparathyroidism, hypothyroidism, hyperthyroidism, and obesity [3,6]. However, when these conditions are identified and treated in a timely manner, they do not appear to play a major role in growth impairment [6,9]. Growth hormone deficiency is considered rare in this syndrome [3].

This study has limitations inherent to the retrospective collection of data from medical records; however, rigorous data processing was undertaken to minimize these effects. Additional limitations include the reduced number of measurements in certain age groups, particularly among males, and the insufficient data available to construct growth charts for individuals aged 17 to 20 years.

Despite these limitations, this study provides Brazilian growth charts for individuals with 22q11.2DS from birth to 17 years of age. These charts represent important tools for more accurate clinical assessment, allowing better adjustment of growth expectations, particularly during the first two years of life, and facilitating recognition of appropriate growth recovery after early childhood. Their use may help reduce unnecessary investigations or delays in the evaluation of growth deviations, thereby improving health care delivery and quality of life for this population. In addition, this study is the first to compare growth charts for individuals with 22q11.2DS with reference population data[12] across the age range of 2 to 17 years. Previous work by Guzmán et al [9]. compared growth data from Chilean children with 22q11.2DS aged birth to 24 months with WHO reference data.

In conclusion, this study presents the first Brazilian sex-specific growth charts for individuals with 22q11.2DS, including weight-for-age, height-for-age, and head circumference–for-age from birth to 2 years, and weight-for-age, height-for-age, and BMI-for-age from 2 to 17 years. These tools enable more realistic comparisons within this population and support individualized care strategies aimed at improving health outcomes and quality of life. It is expected that the use of these charts will assist both families and health care professionals in the clinical management of children and adolescents with 22q11.2DS.

## Funding

This research did not receive any specific funding from public, commercial, or not-for-profit agencies.

## Data availability

The data that support the findings of this study are available from the corresponding author.

## Authors’ contributions

Data collection and manuscript writing.

## Conflicts of interest

The authors declare no conflicts of interest.

## Appendix Supplementary materials

Image, application 1
