# Supplementary material for: Brazilian growth charts for 22q11.2 deletion syndrome from birth to 17 years
Source: J Pediatr (Rio J). 2026 May 30;102(4):101560. doi: 10.1016/j.jped.2026.101560 (PMC13241978; doi:10.1016/j.jped.2026.101560)
Supplement: Supplementary file 1 [file mmc1.docx]

**JPED-D-26-00087_ Supplementary Material**

### **Supplementary Tables**

**Supplementary Table 1.** Total number of 22q11.2DS data excluded according to exclusion criteria 1–7 and data from 18 to 20 years of age.

| **Exclusion criteria** | **Weight (n)** | **Height (n)** | **HC (n)** | **Total (n)** | **Patients (n)** |
| --- | --- | --- | --- | --- | --- |
| *1. Missing age* | 5 | 4 | 2 | 11 | 1 |
| *2. Preterm < 2 years* | 71 | 64 | 52 | 187 | 2 |
| *3. Hypothyroidism* | 0 | 0 | 0 | 0 | 0 |
| *4. Gh deficiency* | 0 | 0 | 0 | 0 | 0 |
| *5. Congenital heart disease* | 0 | 0 | 0 | 0 | 0 |
| *6. Duplicates* | 453 | 377 | 106 | 936 | 0 |
| *7. More than five SD* | 73 | 75 | 18 | 166 | 3 |
| *Data from 18 to 20 years* | 11 | 12 | 0 | 23 | 0 |
| **TOTAL** | **613** | **532** | **178** | **1.323** | **6** |

(n) total number of excluded data.

**Supplementary Table 2.** Height (m) data from birth to 24 months in patients with 22q11.2DS, stratified by sex and age.

|  | **Male** | | | | | **Female** | | | | | | |
| --- | --- | --- | --- | --- | --- | --- | --- | --- | --- | --- | --- | --- |
| **Age (months)** | **n** | **Min** | **Max** | **Mean** | **SD** | | **n** | **Min** | **Max** | **Mean** | **SD** |  |
| Birth | 26 | 0.43 | 0.54 | 0.47 | 0.03 | | 44 | 0.43 | 0.53 | 0.47 | 0.02 |  |
| 1 | 7 | 0.47 | 0.56 | 0.52 | 0.03 | | 9 | 0.47 | 0.61 | 0.52 | 0.04 |  |
| 2 | 8 | 0.52 | 0.64 | 0.57 | 0.04 | | 8 | 0.50 | 0.63 | 0.55 | 0.04 |  |
| 3 | 8 | 0.55 | 0.65 | 0.59 | 0.03 | | 6 | 0.54 | 0.58 | 0.57 | 0.01 |  |
| 4 | 10 | 0.58 | 0.68 | 0.62 | 0.03 | | 7 | 0.54 | 0.61 | 0.57 | 0.02 |  |
| 5 | 7 | 0.59 | 0.66 | 0.62 | 0.02 | | 11 | 0.50 | 0.66 | 0.59 | 0.04 |  |
| 6 | 7 | 0.61 | 0.65 | 0.64 | 0.01 | | 8 | 0.58 | 0.65 | 0.61 | 0.03 |  |
| 7 | 6 | 0.62 | 0.68 | 0.66 | 0.02 | | 7 | 0.58 | 0.65 | 0.62 | 0.03 |  |
| 8 | 7 | 0.63 | 0.70 | 0.67 | 0.02 | | 8 | 0.60 | 0.66 | 0.62 | 0.02 |  |
| 9 | 5 | 0.64 | 0.79 | 0.69 | 0.05 | | 9 | 0.60 | 0.68 | 0.63 | 0.03 |  |
| 10 | 7 | 0.65 | 0.74 | 0.70 | 0.03 | | 6 | 0.63 | 0.69 | 0.66 | 0.02 |  |
| 11 | 8 | 0.67 | 0.75 | 0.71 | 0.03 | | 10 | 0.61 | 0.73 | 0.67 | 0.03 |  |
| 12 | 8 | 0.69 | 0.77 | 0.73 | 0.03 | | 7 | 0.61 | 0.73 | 0.68 | 0.03 |  |
| 13 | 5 | 0.70 | 0.77 | 0.74 | 0.03 | | 5 | 0.66 | 0.75 | 0.70 | 0.03 |  |
| 14 | 4 | 0.70 | 0.78 | 0.74 | 0.03 | | 4 | 0.69 | 0.76 | 0.73 | 0.03 |  |
| 15 | 4 | 0.72 | 0.78 | 0.74 | 0.02 | | 7 | 0.70 | 0.73 | 0.71 | 0.01 |  |
| 16 | 3 | 0.71 | 0.74 | 0.72 | 0.01 | | 4 | 0.70 | 0.76 | 0.73 | 0.02 |  |
| 17 | 5 | 0.74 | 0.81 | 0.77 | 0.03 | | 7 | 0.68 | 0.77 | 0.74 | 0.03 |  |
| 18 | 6 | 0.75 | 0.82 | 0.78 | 0.03 | | 9 | 0.67 | 0.76 | 0.74 | 0.03 |  |
| 19 | 3 | 0.76 | 0.83 | 0.78 | 0.03 | | 5 | 0.73 | 0.78 | 0.75 | 0.02 |  |
| 20 | 3 | 0.76 | 0.79 | 0.77 | 0.01 | | 4 | 0.74 | 0.82 | 0.78 | 0.03 |  |
| 21 | 4 | 0.79 | 0.85 | 0.82 | 0.03 | | 8 | 0.70 | 0.82 | 0.76 | 0.03 |  |
| 22 | 5 | 0.78 | 0.86 | 0.81 | 0.04 | | 3 | 0.76 | 0.78 | 0.77 | 0.01 |  |
| 23 | 6 | 0.79 | 0.87 | 0.81 | 0.03 | | 10 | 0.70 | 0.83 | 0.78 | 0.04 |  |
| 24 | 13 | 0.80 | 0.94 | 0.86 | 0.04 | | 17 | 0.71 | 0.94 | 0.85 | 0.06 |  |

Data are presented as number of observations (n), minimum (Min), maximum (Max), mean, and standard deviation (SD).

**Supplementary Table 3.** Weight (kg) data from birth to 24 months in patients with 22q11.2DS, stratified by sex and age.

|  | **Male** | | | | | **Female** | | | | | |  |
| --- | --- | --- | --- | --- | --- | --- | --- | --- | --- | --- | --- | --- |
| **Age (months)** | **n** | **Min** | **Max** | **Mean** | **SD** | | **n** | **Min** | **Max** | **Mean** | **SD** | |
| Birth | 29 | 2.00 | 4.18 | 3.13 | 0.49 | | 48 | 2.11 | 4.10 | 2.90 | 0.44 | |
| 1 | 8 | 3.07 | 5.10 | 3.97 | 0.59 | | 10 | 2.49 | 5.35 | 3.63 | 0.86 | |
| 2 | 8 | 4.02 | 6.50 | 4.94 | 0.77 | | 7 | 3.65 | 6.21 | 4.26 | 0.82 | |
| 3 | 7 | 4.14 | 6.80 | 5.45 | 0.86 | | 7 | 2.80 | 4.80 | 4.33 | 0.68 | |
| 4 | 9 | 4.58 | 7.31 | 6.06 | 0.96 | | 6 | 3.89 | 5.20 | 4.63 | 0.56 | |
| 5 | 9 | 5.17 | 7.70 | 6.45 | 0.86 | | 12 | 2.40 | 6.43 | 4.78 | 1.18 | |
| 6 | 8 | 5.36 | 7.83 | 6.52 | 0.88 | | 7 | 4.43 | 6.95 | 5.64 | 0.86 | |
| 7 | 6 | 6.15 | 7.96 | 7.04 | 0.85 | | 7 | 3.91 | 7.10 | 5.61 | 0.97 | |
| 8 | 6 | 5.90 | 8.30 | 6.87 | 0.91 | | 8 | 4.07 | 6.55 | 5.31 | 0.87 | |
| 9 | 5 | 6.55 | 8.83 | 7.66 | 0.85 | | 11 | 4.70 | 6.79 | 5.76 | 0.71 | |
| 10 | 6 | 6.80 | 9.08 | 7.95 | 0.87 | | 8 | 4.88 | 7.11 | 5.99 | 0.72 | |
| 11 | 8 | 6.84 | 9.17 | 8.18 | 0.80 | | 12 | 5.50 | 8.29 | 6.88 | 0.86 | |
| 12 | 8 | 7.62 | 10.40 | 8.87 | 0.84 | | 6 | 5.73 | 7.84 | 6.82 | 0.83 | |
| 13 | 5 | 7.82 | 9.74 | 8.94 | 0.74 | | 8 | 6.20 | 8.39 | 7.39 | 0.74 | |
| 14 | 3 | 8.77 | 9.75 | 9.37 | 0.43 | | 6 | 6.68 | 8.44 | 7.80 | 0.55 | |
| 15 | 5 | 8.87 | 10.48 | 9.65 | 0.66 | | 7 | 6.54 | 9.60 | 7.76 | 0.91 | |
| 16 | 4 | 7.82 | 9.78 | 8.53 | 0.74 | | 5 | 6.98 | 8.75 | 7.75 | 0.60 | |
| 17 | 4 | 8.84 | 10.66 | 9.68 | 0.66 | | 7 | 6.80 | 9.10 | 7.98 | 0.75 | |
| 18 | 6 | 9.73 | 11.20 | 10.37 | 0.57 | | 9 | 6.72 | 10.65 | 8.36 | 1.23 | |
| 19 | 2 | 10.00 | 10.10 | 10.05 | 0.05 | | 6 | 7.49 | 9.70 | 8.52 | 0.71 | |
| 20 | 3 | 10.14 | 10.20 | 10.17 | 0.02 | | 5 | 7.63 | 8.86 | 8.29 | 0.54 | |
| 21 | 4 | 9.47 | 11.38 | 10.39 | 0.72 | | 8 | 7.20 | 11.50 | 8.89 | 1.34 | |
| 22 | 5 | 9.50 | 11.54 | 10.38 | 0.71 | | 5 | 8.38 | 11.18 | 9.61 | 1.19 | |
| 23 | 6 | 9.50 | 11.68 | 10.68 | 0.83 | | 11 | 6.95 | 11.20 | 9.20 | 1.27 | |
| 24 | 12 | 9.90 | 13.90 | 11.62 | 1.29 | | 24 | 7.40 | 13.60 | 10.68 | 1.66 | |

Data are presented as number of observations (n), minimum (Min), maximum (Max), mean, and standard deviation (SD).

**Supplementary Table 4.** Head circumference (cm) data from birth to 24 months in patients with 22q11.2DS, stratified by sex and age.

|  | **Male** | | | | | | **Female** | | | | | | |
| --- | --- | --- | --- | --- | --- | --- | --- | --- | --- | --- | --- | --- | --- |
| **Age (months)** | | **n** | **Min** | **Max** | **Mean** | **SD** | | **n** | **Min** | **Max** | **Mean** | **SD** |  |
| Birth | | 18 | 31.00 | 37.00 | 34.40 | 1.74 | | 37 | 29.20 | 35.00 | 33.06 | 1.26 |  |
| 1 | | 7 | 35.50 | 38.00 | 36.99 | 0.78 | | 7 | 31.00 | 37.80 | 35.04 | 2.45 |  |
| 2 | | 2 | 38.10 | 39.00 | 38.55 | 0.45 | | 5 | 36.50 | 38.00 | 37.44 | 0.69 |  |
| 3 | | 6 | 39.00 | 40.60 | 40.02 | 0.52 | | 4 | 36.30 | 39.00 | 37.45 | 0.99 |  |
| 4 | | 5 | 39.50 | 43.00 | 41.30 | 1.21 | | 5 | 36.50 | 41.00 | 38.30 | 1.50 |  |
| 5 | | 6 | 40.50 | 42.60 | 41.68 | 0.71 | | 5 | 37.50 | 42.30 | 40.26 | 1.76 |  |
| 6 | | 4 | 40.50 | 43.00 | 41.75 | 1.03 | | 5 | 39.00 | 43.00 | 40.40 | 1.39 |  |
| 7 | | 6 | 41.00 | 44.50 | 43.17 | 1.18 | | 5 | 38.00 | 43.00 | 40.96 | 1.88 |  |
| 8 | | 5 | 42.00 | 44.50 | 43.48 | 0.95 | | 6 | 39.00 | 45.00 | 41.33 | 2.30 |  |
| 9 | | 3 | 43.50 | 45.00 | 44.33 | 0.62 | | 6 | 39.50 | 45.00 | 42.05 | 2.05 |  |
| 10 | | 6 | 44.00 | 45.60 | 44.85 | 0.50 | | 5 | 40.50 | 45.00 | 42.50 | 1.73 |  |
| 11 | | 5 | 44.00 | 46.00 | 45.10 | 0.92 | | 7 | 40.50 | 45.00 | 43.11 | 1.71 |  |
| 12 | | 4 | 45.50 | 46.50 | 46.00 | 0.35 | | 5 | 42.00 | 45.00 | 43.04 | 1.03 |  |
| 13 | | 4 | 44.50 | 46.60 | 45.58 | 0.79 | | 4 | 41.00 | 47.00 | 43.55 | 2.26 |  |
| 14 | | 1 | 47.00 | 47.00 | 47.00 | 0.00 | | 2 | 42.50 | 47.00 | 44.75 | 2.25 |  |
| 15 | | 4 | 46.50 | 47.30 | 47.03 | 0.33 | | 3 | 43.70 | 45.00 | 44.40 | 0.54 |  |
| 16 | | 3 | 44.50 | 47.70 | 45.90 | 1.34 | | 0 | – | – | – | – |  |
| 17 | | 1 | 47.50 | 47.50 | 47.50 | 0.00 | | 3 | 44.00 | 48.00 | 46.17 | 1.65 |  |
| 18 | | 3 | 47.50 | 48.00 | 47.80 | 0.22 | | 3 | 42.00 | 44.00 | 43.33 | 0.94 |  |
| 19 | | 2 | 46.50 | 48.00 | 47.25 | 0.75 | | 1 | 48.00 | 48.00 | 48.00 | 0.00 |  |
| 20 | | 1 | 49.00 | 49.00 | 49.00 | 0.00 | | 2 | 43.50 | 46.00 | 44.75 | 1.25 |  |
| 21 | | 3 | 48.30 | 49.00 | 48.60 | 0.29 | | 3 | 44.50 | 48.00 | 45.83 | 1.55 |  |
| 22 | | 2 | 48.80 | 48.90 | 48.85 | 0.05 | | 2 | 44.00 | 44.60 | 44.30 | 0.30 |  |
| 23 | | 0 | – | – | – | – | | 4 | 40.00 | 45.00 | 43.00 | 1.87 |  |
| 24 | | 5 | 44.00 | 49.00 | 47.08 | 1.83 | | 7 | 44.50 | 48.00 | 45.51 | 1.14 |  |

Data are presented as number of observations (n), minimum (Min), maximum (Max), mean, and standard deviation (SD).

**Supplementary Table 5.** Height (m) data from 2 to 17 years of age in patients with 22q11.2DS, stratified by sex and age.

|  | | **Male** | | | | | **Female** | | | | | |  |
| --- | --- | --- | --- | --- | --- | --- | --- | --- | --- | --- | --- | --- | --- |
| **Age (years)** | **n** | | **Min** | **Max** | **Mean** | **SD** | | **n** | **Min** | **Max** | **Mean** | **SD** | |
| 2 | 19 | | 0.71 | 0.94 | 0.85 | 0.06 | | 17 | 0.80 | 1.04 | 0.87 | 0.06 | |
| 3 | 18 | | 0.77 | 1.00 | 0.92 | 0.05 | | 16 | 0.88 | 1.04 | 0.95 | 0.06 | |
| 4 | 20 | | 0.84 | 1.04 | 0.96 | 0.05 | | 10 | 0.91 | 1.08 | 1.00 | 0.05 | |
| 5 | 20 | | 0.86 | 1.11 | 1.02 | 0.06 | | 11 | 0.96 | 1.12 | 1.07 | 0.05 | |
| 6 | 10 | | 0.94 | 1.26 | 1.13 | 0.08 | | 9 | 1.03 | 1.30 | 1.16 | 0.07 | |
| 7 | 15 | | 0.99 | 1.28 | 1.20 | 0.07 | | 9 | 1.06 | 1.32 | 1.18 | 0.07 | |
| 8 | 19 | | 1.10 | 1.40 | 1.25 | 0.07 | | 9 | 1.11 | 1.35 | 1.23 | 0.07 | |
| 9 | 17 | | 1.20 | 1.41 | 1.28 | 0.06 | | 9 | 1.16 | 1.53 | 1.29 | 0.10 | |
| 10 | 12 | | 1.27 | 1.52 | 1.38 | 0.08 | | 5 | 1.20 | 1.38 | 1.28 | 0.06 | |
| 11 | 12 | | 1.33 | 1.52 | 1.42 | 0.07 | | 5 | 1.24 | 1.55 | 1.36 | 0.10 | |
| 12 | 8 | | 1.28 | 1.56 | 1.45 | 0.08 | | 7 | 1.26 | 1.78 | 1.51 | 0.15 | |
| 13 | 9 | | 1.45 | 1.64 | 1.54 | 0.06 | | 6 | 1.48 | 1.81 | 1.57 | 0.11 | |
| 14 | 8 | | 1.22 | 1.63 | 1.50 | 0.12 | | 3 | 1.55 | 1.62 | 1.58 | 0.03 | |
| 15 | 7 | | 1.40 | 1.65 | 1.56 | 0.09 | | 5 | 1.54 | 1.81 | 1.63 | 0.10 | |
| 16 | 5 | | 1.37 | 1.66 | 1.55 | 0.10 | | 5 | 1.57 | 1.68 | 1.63 | 0.04 | |
| 17 | 3 | | 1.40 | 1.59 | 1.51 | 0.08 | | 2 | 1.58 | 1.63 | 1.61 | 0.02 | |

Data are presented as number of observations (n), minimum (Min), maximum (Max), mean, and standard deviation (SD).

**Supplementary Table 6.** Weight (kg) data from 2 to 17 years of age in patients with 22q11.2DS, stratified by sex and age.

|  | **Male** | | | | | **Female** | | | | | | |
| --- | --- | --- | --- | --- | --- | --- | --- | --- | --- | --- | --- | --- |
| **Age (years)** | **n** | **Min** | **Max** | **Mean** | **SD** | | **n** | **Min** | **Max** | **Mean** | **SD** |  |
| 2 | 16 | 9.9 | 13.90 | 11.91 | 1.27 | | 25 | 7.4 | 13.60 | 10.66 | 1.64 |  |
| 3 | 16 | 11.85 | 18.00 | 14.19 | 1.73 | | 21 | 8.15 | 17.00 | 12.64 | 2.24 |  |
| 4 | 11 | 13.0 | 17.80 | 15.42 | 1.43 | | 22 | 9.19 | 18.00 | 13.91 | 2.17 |  |
| 5 | 11 | 15.0 | 21.10 | 17.91 | 1.51 | | 20 | 11.0 | 19.60 | 15.20 | 2.18 |  |
| 6 | 9 | 16.8 | 25.00 | 21.45 | 2.60 | | 14 | 12.0 | 29.50 | 20.28 | 4.83 |  |
| 7 | 10 | 19.0 | 28.80 | 22.86 | 3.10 | | 17 | 13.3 | 37.50 | 23.30 | 6.43 |  |
| 8 | 10 | 20.7 | 38.00 | 28.28 | 6.07 | | 20 | 16.3 | 45.00 | 27.26 | 7.70 |  |
| 9 | 9 | 20.0 | 47.00 | 27.78 | 7.41 | | 20 | 16.8 | 45.50 | 29.35 | 8.36 |  |
| 10 | 5 | 23.8 | 34.00 | 28.03 | 3.53 | | 13 | 20.5 | 50.00 | 34.65 | 8.51 |  |
| 11 | 5 | 27.8 | 52.30 | 34.05 | 9.19 | | 13 | 21.59 | 55.30 | 36.22 | 9.27 |  |
| 12 | 6 | 31.8 | 57.20 | 45.03 | 10.28 | | 7 | 27.3 | 45.45 | 36.95 | 5.56 |  |
| 13 | 5 | 36.8 | 65.50 | 46.26 | 10.35 | | 7 | 36.0 | 54.20 | 44.33 | 6.57 |  |
| 14 | 5 | 40.2 | 68.60 | 54.12 | 11.60 | | 8 | 31.25 | 54.00 | 44.59 | 7.16 |  |
| 15 | 4 | 43.4 | 71.20 | 54.03 | 10.89 | | 7 | 35.0 | 66.50 | 54.71 | 9.86 |  |
| 16 | 5 | 45.0 | 73.00 | 56.12 | 9.92 | | 5 | 51.8 | 70.10 | 60.18 | 6.63 |  |
| 17 | 2 | 45.4 | 52.00 | 48.70 | 3.30 | | 3 | 62.0 | 73.40 | 66.13 | 5.15 |  |

Data are presented as number of observations (n), minimum (Min), maximum (Max), mean, and standard deviation (SD).

**Supplementary Table 7.** Body mass index (BMI, kg/m²) data from 2 to 17 years of age in patients with 22q11.2DS, stratified by sex and age.

|  | **Male** | | | | | **Female** | | | | |
| --- | --- | --- | --- | --- | --- | --- | --- | --- | --- | --- |
| **Age (years)** | **n** | **Min** | **Max** | **Mean** | **SD** | **n** | **Min** | **Max** | **Mean** | **SD** |
| 2 | 16 | 12.34 | 17.57 | 15.69 | 1.35 | 19 | 13.38 | 16.93 | 15.13 | 0.98 |
| 3 | 16 | 13.59 | 18.74 | 15.83 | 1.20 | 18 | 12.78 | 18.00 | 14.87 | 1.20 |
| 4 | 10 | 13.48 | 17.99 | 15.50 | 1.42 | 20 | 13.00 | 18.64 | 15.14 | 1.34 |
| 5 | 11 | 14.04 | 16.82 | 15.67 | 0.92 | 20 | 13.03 | 18.59 | 14.98 | 1.40 |
| 6 | 9 | 13.65 | 17.44 | 15.87 | 1.16 | 11 | 12.73 | 20.49 | 15.88 | 2.09 |
| 7 | 9 | 13.44 | 17.42 | 16.52 | 1.13 | 13 | 12.93 | 18.35 | 15.57 | 1.48 |
| 8 | 7 | 14.69 | 20.85 | 17.44 | 1.93 | 16 | 12.36 | 21.89 | 17.04 | 2.11 |
| 9 | 9 | 13.44 | 20.08 | 16.51 | 2.05 | 13 | 12.82 | 19.33 | 16.40 | 2.04 |
| 10 | 5 | 14.53 | 18.66 | 17.00 | 1.38 | 10 | 12.61 | 19.08 | 16.87 | 1.77 |
| 11 | 3 | 16.27 | 20.06 | 17.55 | 1.77 | 10 | 12.82 | 20.93 | 17.90 | 2.37 |
| 12 | 6 | 17.15 | 21.99 | 19.97 | 1.64 | 8 | 15.98 | 21.03 | 18.43 | 1.61 |
| 13 | 4 | 16.36 | 20.75 | 18.16 | 1.63 | 7 | 16.35 | 24.77 | 20.23 | 2.43 |
| 14 | 2 | 16.73 | 17.95 | 17.34 | 0.61 | 7 | 17.51 | 21.63 | 19.83 | 1.37 |
| 15 | 3 | 17.31 | 22.95 | 19.56 | 2.44 | 5 | 17.86 | 25.03 | 21.94 | 2.89 |
| 16 | 4 | 18.26 | 21.74 | 19.56 | 1.34 | 4 | 19.38 | 25.59 | 23.10 | 2.28 |
| 17 | 2 | 18.19 | 19.57 | 18.88 | 0.69 | 2 | 24.92 | 26.14 | 25.53 | 0.61 |

Data are presented as number of observations (n), minimum (Min), maximum (Max), mean, and standard deviation (SD).

### **Supplementary Figures**


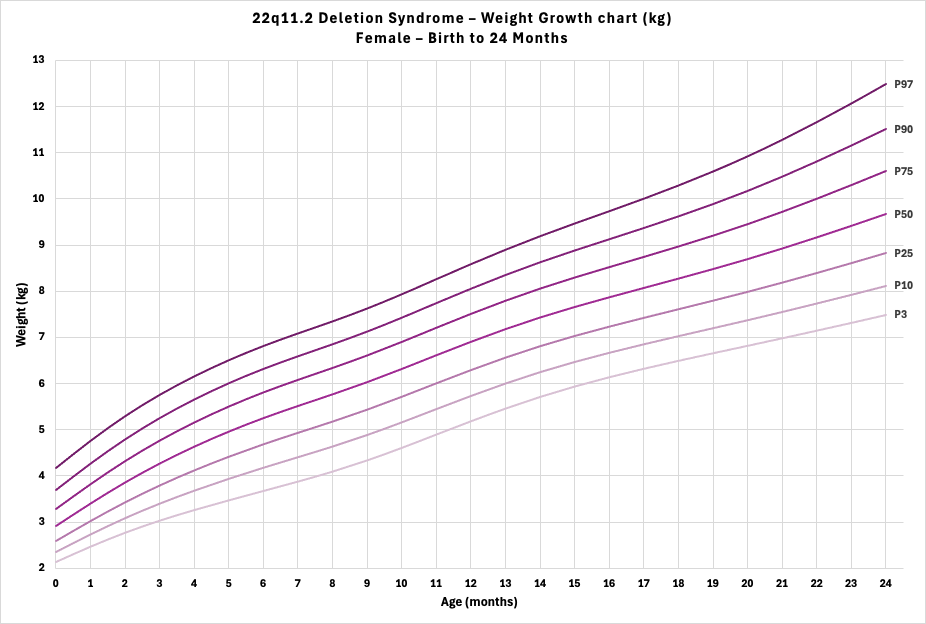


**Supplementary Figure 1.** Weight-for-age growth chart for females with 22q11.2DS from birth to 24 months.


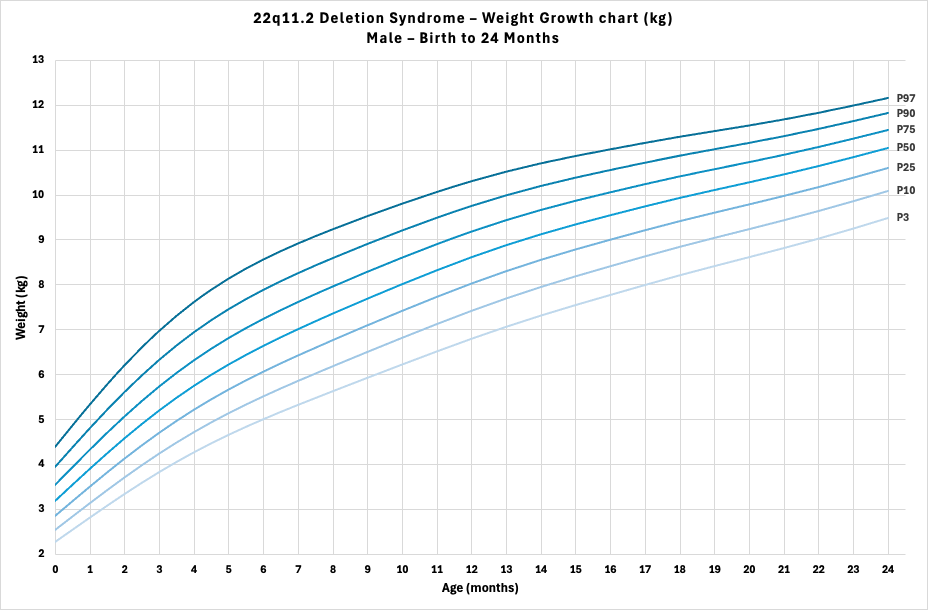


**Supplementary Figure 2.** Weight-for-age growth chart for males with 22q11.2DS from birth to 24 months.


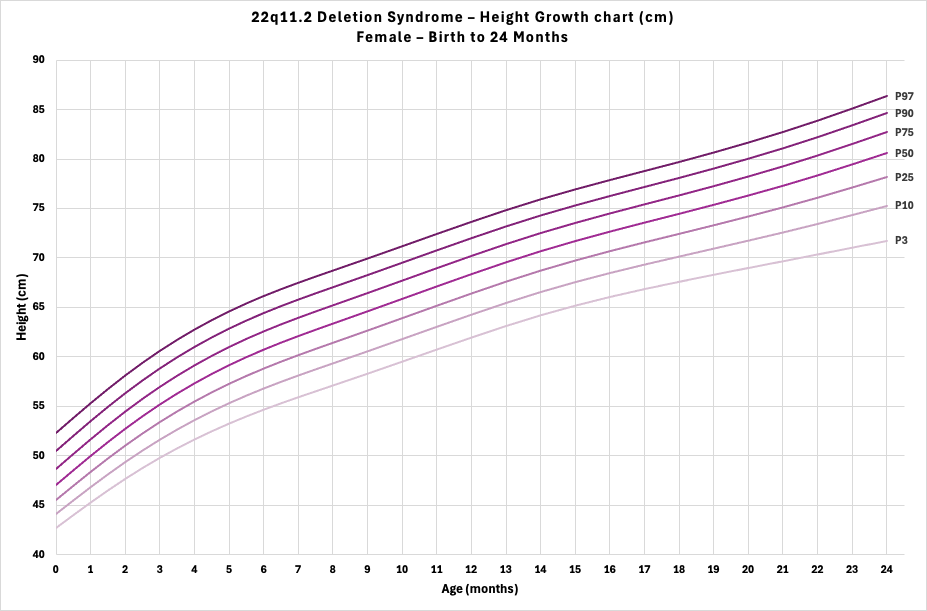


**Supplementary Figure 3.** Height-for-age growth chart for females with 22q11.2DS from birth to 24 months.


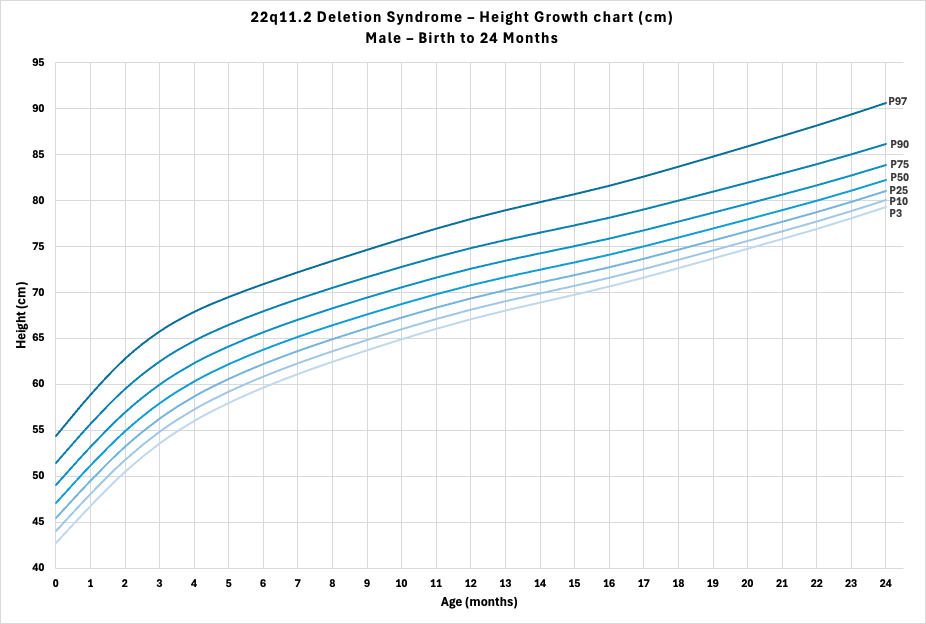


**Supplementary Figure 4.** Height-for-age growth chart for males with 22q11.2DS from birth to 24 months.


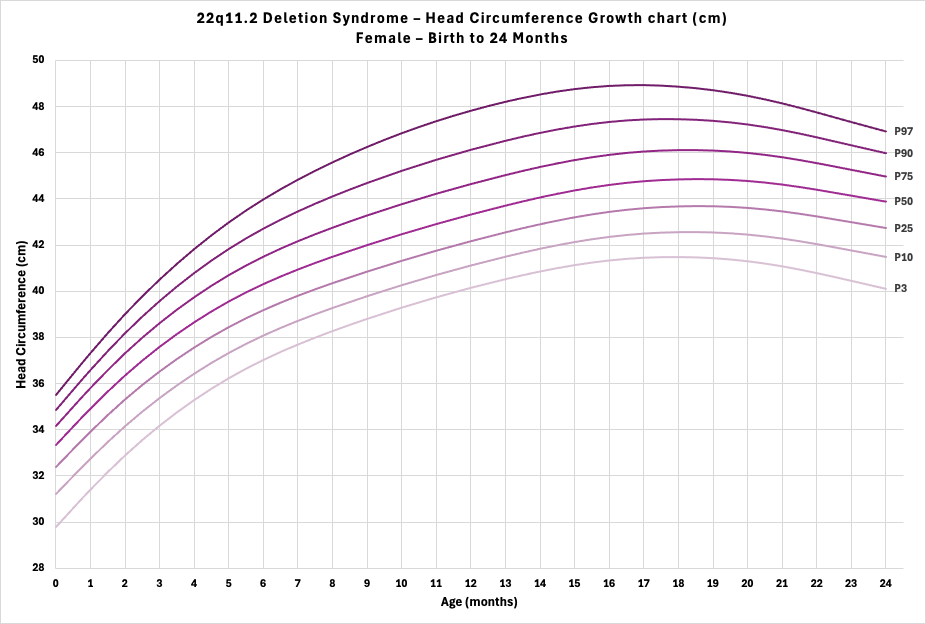


**Supplementary Figure 5.** Head circumference–for-age growth chart for females with 22q11.2DS from birth to 24 months.


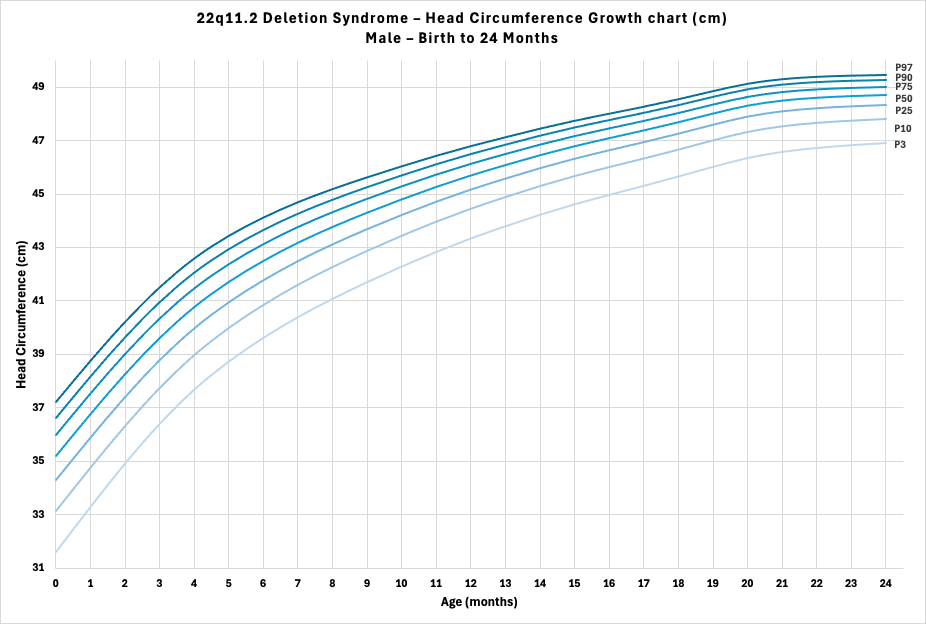


**Supplementary Figure 6.** Head circumference–for-age growth chart for males with 22q11.2DS from birth to 24 months.

**
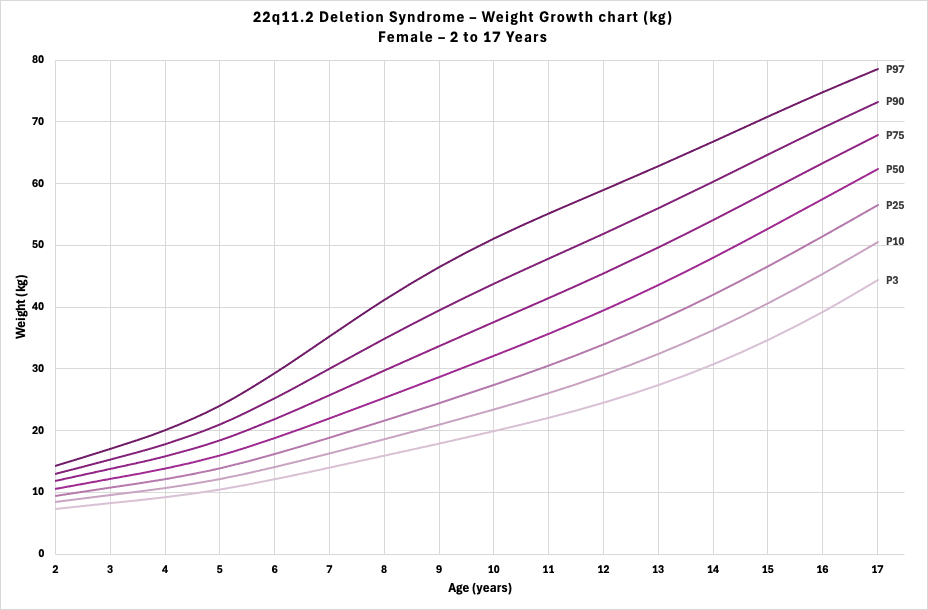
**

**Supplementary Figure 7.** Weight-for-age growth chart for females with 22q11.2DS from 2 to 17 years of age.

.


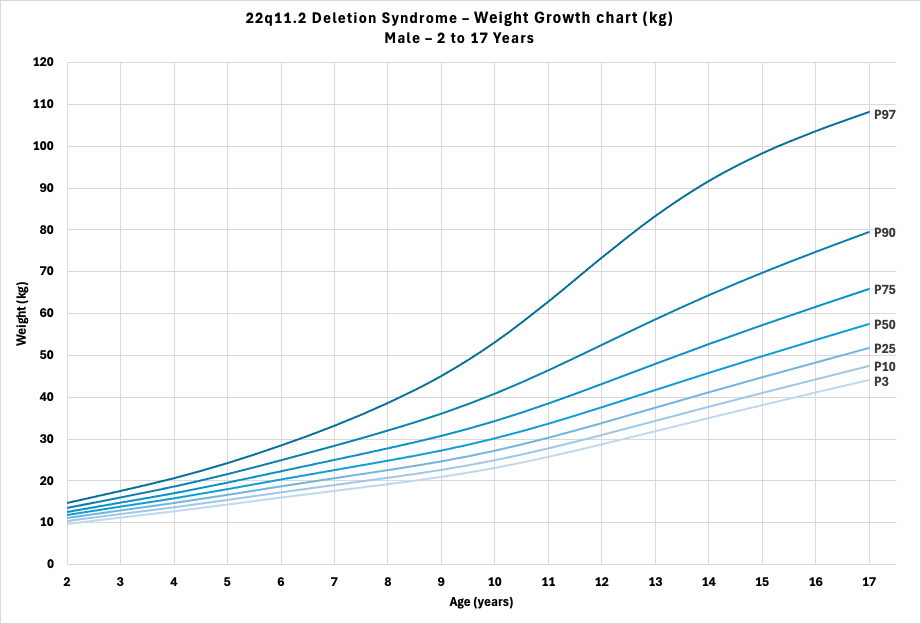


**Supplementary Figure 8.** Weight-for-age growth chart for males with 22q11.2DS from 2 to 17 years of age.


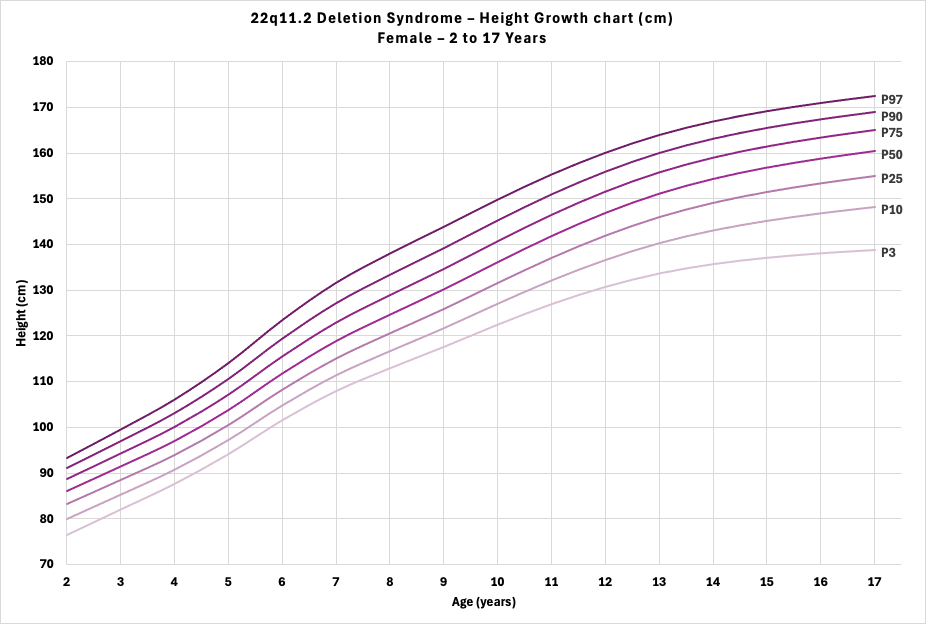


**Supplementary Figure 9.** Height-for-age growth chart for females with 22q11.2DS from 2 to 17 years of age.


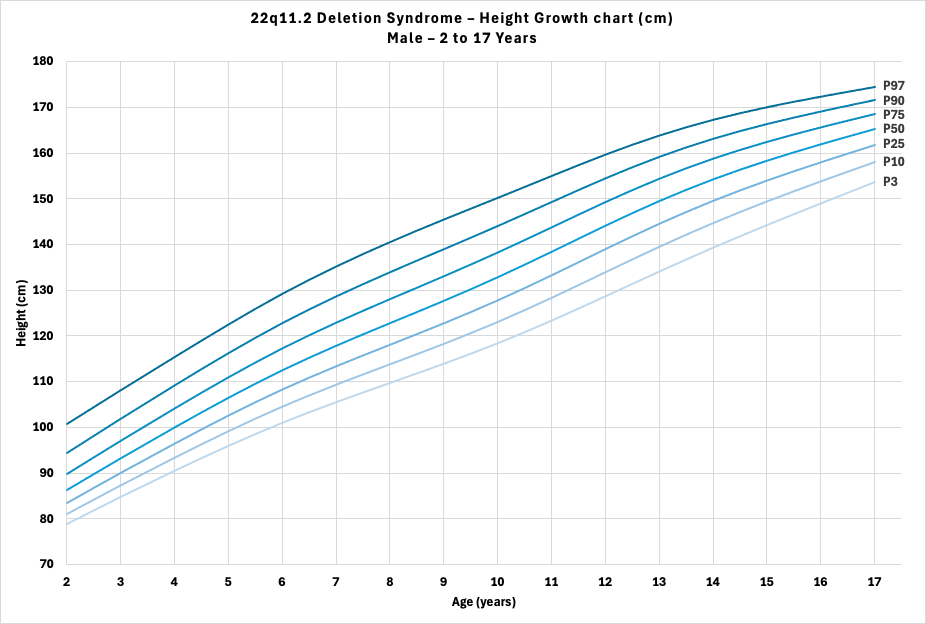


**Supplementary Figure 10.** Height-for-age growth chart for males with 22q11.2DS from 2 to 17 years of age.


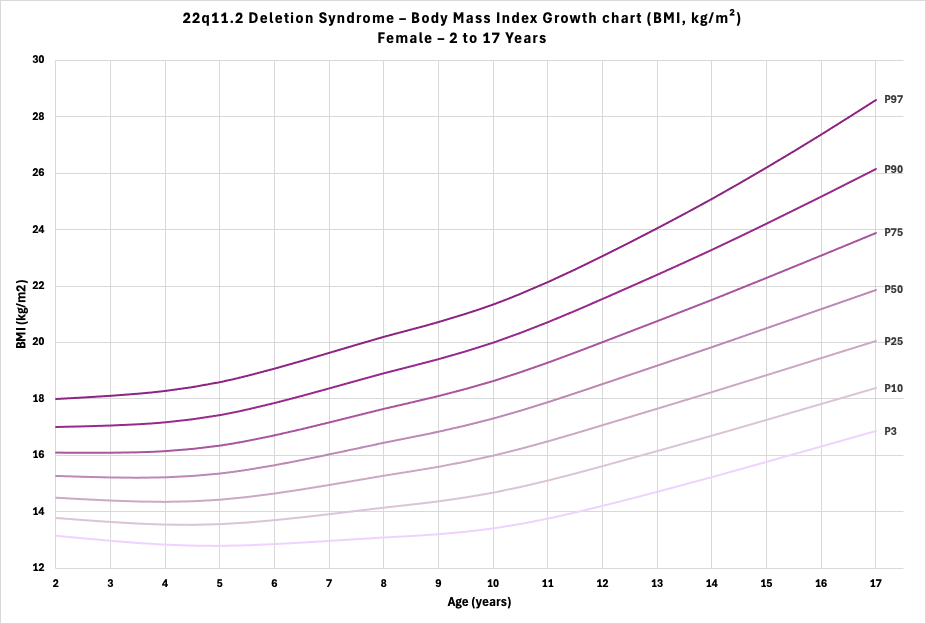


**Supplementary Figure 11.** Body mass index–for-age growth chart for females with 22q11.2DS from 2 to 17 years of age.


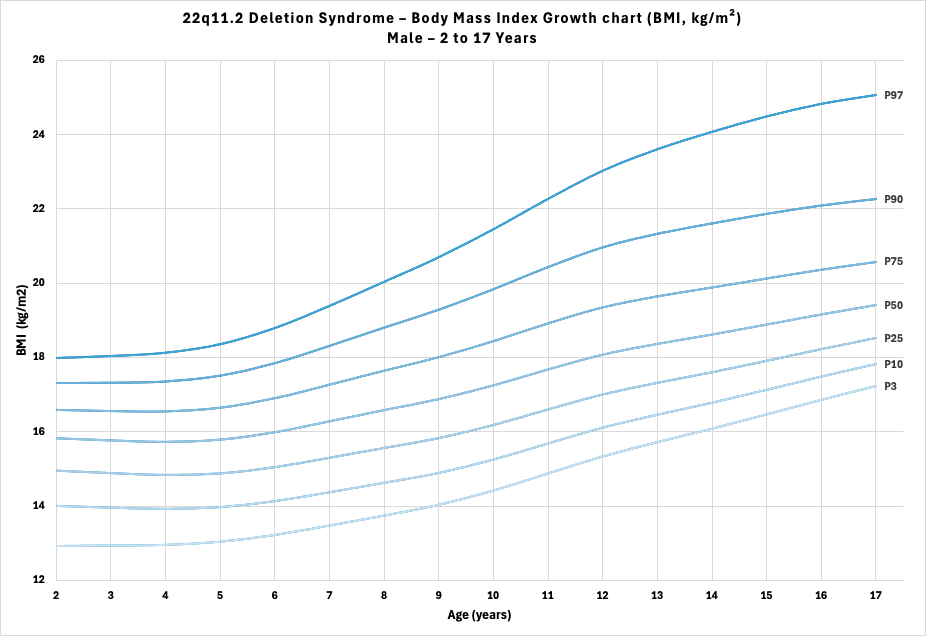


**Supplementary Figure 12.** Body mass index–for-age growth chart for males with 22q11.2DS from 2 to 17 years of age.

### **Supplementary LMS Tables**

**Supplementary Table 8.** LMS parameters and weight percentiles (3rd, 10th, 25th, 50th, 75th, 90th, and 97th) for females with 22q11.2DS from birth to 24 months.

|  | | | | **Percentiles (Weight, kg), Female** | | | | | | |
| --- | --- | --- | --- | --- | --- | --- | --- | --- | --- | --- |
| **Age** | **L** | **M** | **S** | **P3** | **P10** | **P25** | **P50** | **P75** | **P90** | **P97** |
| 0 | -0,490807 | 2,904632 | 0,175788 | 2,138109 | 2,345813 | 2,588455 | 2,904632 | 3,282035 | 3,687852 | 4,166435 |
| 1 | -0,196368 | 3,391237 | 0,173876 | 2,470057 | 2,726725 | 3,019971 | 3,391237 | 3,818447 | 4,259058 | 4,754978 |
| 2 | 0,096587 | 3,848054 | 0,171748 | 2,771527 | 3,080495 | 3,424901 | 3,848054 | 4,317889 | 4,784420 | 5,289129 |
| 3 | 0,378227 | 4,258447 | 0,169273 | 3,033379 | 3,395852 | 3,789346 | 4,258447 | 4,762061 | 5,245628 | 5,751812 |
| 4 | 0,631052 | 4,623936 | 0,166361 | 3,263115 | 3,677642 | 4,115937 | 4,623936 | 5,153420 | 5,647845 | 6,152098 |
| 5 | 0,840534 | 4,950707 | 0,162926 | 3,473657 | 3,935105 | 4,411555 | 4,950707 | 5,499403 | 6,000859 | 6,502505 |
| 6 | 0,996899 | 5,243116 | 0,158890 | 3,677079 | 4,175846 | 4,681310 | 5,243116 | 5,805109 | 6,311065 | 6,810627 |
| 7 | 1,103399 | 5,506325 | 0,154348 | 3,880479 | 4,405007 | 4,929861 | 5,506325 | 6,076603 | 6,585160 | 7,083209 |
| 8 | 1,164396 | 5,758627 | 0,149472 | 4,096628 | 4,636481 | 5,173018 | 5,758627 | 6,334587 | 6,845676 | 7,344094 |
| 9 | 1,184192 | 6,022954 | 0,144474 | 4,339223 | 4,886960 | 5,430524 | 6,022954 | 6,604819 | 7,120530 | 7,622928 |
| 10 | 1,168682 | 6,308240 | 0,139585 | 4,610298 | 5,161232 | 5,709403 | 6,308240 | 6,897622 | 7,420887 | 7,931370 |
| 11 | 1,123550 | 6,605767 | 0,134997 | 4,898963 | 5,449739 | 6,000767 | 6,605767 | 7,203986 | 7,737183 | 8,259091 |
| 12 | 1,054202 | 6,896870 | 0,130867 | 5,186795 | 5,734556 | 6,286586 | 6,896870 | 7,504237 | 8,048609 | 8,584017 |
| 13 | 0,965573 | 7,171991 | 0,127350 | 5,461841 | 6,004951 | 6,556880 | 7,171991 | 7,788926 | 8,345634 | 8,896428 |
| 14 | 0,862656 | 7,423921 | 0,124577 | 5,714213 | 6,252212 | 6,803796 | 7,423921 | 8,051250 | 8,621684 | 9,189904 |
| 15 | 0,751018 | 7,651471 | 0,122632 | 5,939483 | 6,473154 | 7,025198 | 7,651471 | 8,290782 | 8,876904 | 9,465089 |
| 16 | 0,636910 | 7,861588 | 0,121543 | 6,140673 | 6,672175 | 7,226763 | 7,861588 | 8,515600 | 9,120293 | 9,731832 |
| 17 | 0,525349 | 8,064228 | 0,121309 | 6,324345 | 6,856916 | 7,417232 | 8,064228 | 8,736850 | 9,364057 | 10,003390 |
| 18 | 0,420645 | 8,266661 | 0,121865 | 6,496177 | 7,033530 | 7,603280 | 8,266661 | 8,962399 | 9,616597 | 10,288700 |
| 19 | 0,326245 | 8,473230 | 0,123107 | 6,660278 | 7,206179 | 7,789153 | 8,473230 | 9,196670 | 9,882381 | 10,592250 |
| 20 | 0,242650 | 8,689127 | 0,124968 | 6,821402 | 7,379639 | 7,979765 | 8,689127 | 9,445247 | 10,167450 | 10,920630 |
| 21 | 0,169415 | 8,918608 | 0,127333 | 6,984265 | 7,558392 | 8,179446 | 8,918608 | 9,712420 | 10,476220 | 11,278470 |
| 22 | 0,105017 | 9,160214 | 0,130037 | 7,149916 | 7,742654 | 8,387599 | 9,160214 | 9,995919 | 10,805740 | 11,662220 |
| 23 | 0,045647 | 9,410336 | 0,132945 | 7,317935 | 7,930900 | 8,601654 | 9,410336 | 10,291270 | 11,150960 | 12,066530 |
| 24 | -0,012917 | 9,667218 | 0,135937 | 7,489429 | 8,123216 | 8,820749 | 9,667218 | 10,596070 | 11,509200 | 12,488810 |

**Supplementary Table 9.** LMS parameters and weight percentiles (3rd, 10th, 25th, 50th, 75th, 90th, and 97th) for males with 22q11.2DS from birth to 24 months.

|  | | | | **Percentiles (Weight, kg), Male** | | | | | | |
| --- | --- | --- | --- | --- | --- | --- | --- | --- | --- | --- |
| **Age** | **L** | **M** | **S** | **P3** | **P10** | **P25** | **P50** | **P75** | **P90** | **P97** |
| 0 | 0,1274144 | 3,17744 | 0,1660742 | 2,262968 | 2,538183 | 2,842164 | 3,17744 | 3,546722 | 3,952913 | 4,399118 |
| 1 | 0,1107772 | 3,892936 | 0,1608918 | 2,805238 | 3,133154 | 3,494725 | 3,892936 | 4,330998 | 4,812358 | 5,340719 |
| 2 | 0,1096685 | 4,575385 | 0,1556791 | 3,333012 | 3,708802 | 4,121859 | 4,575385 | 5,072814 | 5,617831 | 6,214381 |
| 3 | 0,1355852 | 5,197299 | 0,1503977 | 3,82295 | 4,241097 | 4,698208 | 5,197299 | 5,741577 | 6,334441 | 6,979499 |
| 4 | 0,1909892 | 5,746716 | 0,1450194 | 4,264102 | 4,719007 | 5,212399 | 5,746716 | 6,324501 | 6,948405 | 7,621191 |
| 5 | 0,274994 | 6,223358 | 0,1395389 | 4,654928 | 5,14137 | 5,663649 | 6,223358 | 6,822119 | 7,46158 | 8,143415 |
| 6 | 0,3848578 | 6,638968 | 0,133985 | 5,003429 | 5,517154 | 6,062105 | 6,638968 | 7,248418 | 7,89112 | 8,567732 |
| 7 | 0,5167366 | 7,012552 | 0,128394 | 5,323799 | 5,861761 | 6,424704 | 7,012552 | 7,625233 | 8,262676 | 8,924815 |
| 8 | 0,6657408 | 7,360198 | 0,1228018 | 5,628774 | 6,188614 | 6,765926 | 7,360198 | 7,970964 | 8,597794 | 9,240289 |
| 9 | 0,8269899 | 7,694304 | 0,117244 | 5,928647 | 6,508286 | 7,097005 | 7,694304 | 8,299741 | 8,912921 | 9,533492 |
| 10 | 0,9975064 | 8,017139 | 0,1117771 | 6,225326 | 6,82247 | 7,419744 | 8,017139 | 8,614645 | 9,212255 | 9,809961 |
| 11 | 1,175198 | 8,327192 | 0,1064578 | 6,517366 | 7,129409 | 7,732369 | 8,327192 | 8,914656 | 9,495408 | 10,07 |
| 12 | 1,359077 | 8,620034 | 0,1013331 | 6,800597 | 7,424617 | 8,030323 | 8,620034 | 9,195589 | 9,75848 | 10,30994 |
| 13 | 1,549472 | 8,888285 | 9,64E-02 | 7,068121 | 7,700885 | 8,306258 | 8,888285 | 9,450069 | 9,994065 | 10,52225 |
| 14 | 1,74448 | 9,131451 | 9,18E-02 | 7,318688 | 7,956989 | 8,559236 | 9,131451 | 9,678124 | 10,20271 | 10,70794 |
| 15 | 1,942263 | 9,352183 | 8,74E-02 | 7,553845 | 8,194665 | 8,791394 | 9,352183 | 9,882936 | 10,38809 | 10,87109 |
| 16 | 2,141793 | 9,55679 | 8,32E-02 | 7,77877 | 8,41935 | 9,008587 | 9,55679 | 10,07124 | 10,5573 | 11,01906 |
| 17 | 2,342614 | 9,754883 | 7,93E-02 | 8,001513 | 8,639563 | 9,219882 | 9,754883 | 10,25313 | 10,72081 | 11,1626 |
| 18 | 2,546912 | 9,94383 | 7,55E-02 | 8,218153 | 8,851964 | 9,422375 | 9,94383 | 10,42609 | 10,8761 | 11,29901 |
| 19 | 2,756581 | 10,11966 | 7,20E-02 | 8,42347 | 9,051929 | 9,611847 | 10,11966 | 10,58627 | 11,01929 | 11,42433 |
| 20 | 2,97206 | 10,29145 | 6,88E-02 | 8,623424 | 9,246663 | 9,796524 | 10,29145 | 10,74342 | 11,16072 | 11,54932 |
| 21 | 3,193158 | 10,46848 | 6,58E-02 | 8,825871 | 9,444601 | 9,985315 | 10,46848 | 10,90715 | 11,3102 | 11,684 |
| 22 | 3,41898 | 10,65518 | 6,30E-02 | 9,036994 | 9,651167 | 10,18309 | 10,65518 | 11,08148 | 11,47143 | 11,83172 |
| 23 | 3,647673 | 10,85321 | 6,02E-02 | 9,261335 | 9,869599 | 10,39219 | 10,85321 | 11,26752 | 11,64502 | 11,99264 |
| 24 | 3,876931 | 11,0582 | 5,75E-02 | 9,497657 | 10,09725 | 10,60899 | 11,0582 | 11,4603 | 11,82548 | 12,16082 |

**Supplementary Table 10.** LMS parameters and height percentiles (3rd, 10th, 25th, 50th, 75th, 90th, and 97th) for females with 22q11.2DS from birth to 24 months.

|  | | | | **Percentiles (Height, cm), Female** | | | | | | |
| --- | --- | --- | --- | --- | --- | --- | --- | --- | --- | --- |
| **Age** | **L** | **M** | **S** | **P3** | **P10** | **P25** | **P50** | **P75** | **P90** | **P97** |
| 0 | -0,77143 | 47,08605 | 0,05033 | 42,73525 | 44,10366 | 45,55166 | 47,08605 | 48,71445 | 50,44533 | 52,28823 |
| 1 | -0,14872 | 50,01371 | 0,04980 | 45,30511 | 46,81565 | 48,38434 | 50,01371 | 51,70642 | 53,46527 | 55,29321 |
| 2 | 0,45154 | 52,75816 | 0,04926 | 47,70008 | 49,35506 | 51,04105 | 52,75816 | 54,50647 | 56,28609 | 58,09712 |
| 3 | 1,00745 | 55,21060 | 0,04870 | 49,83065 | 51,62443 | 53,41774 | 55,21060 | 57,00303 | 58,79504 | 60,58664 |
| 4 | 1,49985 | 57,34280 | 0,04811 | 51,68317 | 53,60273 | 55,48852 | 57,34280 | 59,16758 | 60,96465 | 62,73561 |
| 5 | 1,91625 | 59,17750 | 0,04746 | 53,29173 | 55,31739 | 57,27724 | 59,17750 | 61,02341 | 62,81953 | 64,56978 |
| 6 | 2,24887 | 60,74711 | 0,04672 | 54,69827 | 56,80424 | 58,81694 | 60,74711 | 62,60356 | 64,39368 | 66,12372 |
| 7 | 2,50803 | 62,11492 | 0,04592 | 55,95809 | 58,12035 | 60,16764 | 62,11492 | 63,97424 | 65,75546 | 67,46672 |
| 8 | 2,70949 | 63,37298 | 0,04509 | 57,14669 | 59,34726 | 61,41651 | 63,37298 | 65,23130 | 67,00327 | 68,69855 |
| 9 | 2,87204 | 64,61254 | 0,04427 | 58,33745 | 60,56576 | 62,65032 | 64,61254 | 66,46912 | 68,23340 | 69,91617 |
| 10 | 3,00986 | 65,86805 | 0,04346 | 59,55465 | 61,80479 | 63,90133 | 65,86805 | 67,72334 | 69,48174 | 71,15498 |
| 11 | 3,13459 | 67,12491 | 0,04266 | 60,77959 | 63,04807 | 65,15455 | 67,12491 | 68,97900 | 70,73240 | 72,39764 |
| 12 | 3,25739 | 68,36324 | 0,04190 | 61,98981 | 64,27496 | 66,39013 | 68,36324 | 70,21555 | 71,96368 | 73,62088 |
| 13 | 3,38753 | 69,56001 | 0,04117 | 63,15862 | 65,46092 | 67,58465 | 69,56001 | 71,40983 | 73,15182 | 74,80005 |
| 14 | 3,53696 | 70,67908 | 0,04051 | 64,24361 | 66,56696 | 68,70105 | 70,67908 | 72,52586 | 74,26048 | 75,89800 |
| 15 | 3,70988 | 71,70233 | 0,03995 | 65,21829 | 67,57027 | 69,71923 | 71,70233 | 73,54704 | 75,27424 | 76,90026 |
| 16 | 3,90304 | 72,65736 | 0,03956 | 66,09719 | 68,49052 | 70,66306 | 72,65736 | 74,50433 | 76,22719 | 77,84389 |
| 17 | 4,10982 | 73,56567 | 0,03935 | 66,89421 | 69,34455 | 71,55186 | 73,56567 | 75,42133 | 77,14497 | 78,75655 |
| 18 | 4,32290 | 74,45379 | 0,03932 | 67,63033 | 70,15562 | 72,41060 | 74,45379 | 76,32603 | 78,05702 | 79,66911 |
| 19 | 4,53828 | 75,36201 | 0,03949 | 68,33954 | 70,96067 | 73,27815 | 75,36201 | 77,25990 | 79,00587 | 80,62510 |
| 20 | 4,76141 | 76,30240 | 0,03983 | 69,02947 | 71,77061 | 74,16655 | 76,30240 | 78,23449 | 80,00217 | 81,63409 |
| 21 | 4,99810 | 77,28357 | 0,04031 | 69,70664 | 72,59470 | 75,08525 | 77,28357 | 79,25704 | 81,05161 | 82,70009 |
| 22 | 5,24815 | 78,33089 | 0,04089 | 70,39288 | 73,45792 | 76,05989 | 78,33089 | 80,35241 | 82,17846 | 83,84682 |
| 23 | 5,51001 | 79,44217 | 0,04156 | 71,08343 | 74,35872 | 77,08899 | 79,44217 | 81,51746 | 83,37868 | 85,06945 |
| 24 | 5,77828 | 80,58973 | 0,04229 | 71,75609 | 75,27445 | 78,14742 | 80,58973 | 82,72240 | 84,62077 | 86,33509 |

**Supplementary Table 11.** LMS parameters and height percentiles (3rd, 10th, 25th, 50th, 75th, 90th, and 97th) for males with 22q11.2DS from birth to 24 months.

|  | | | | **Percentiles (Height, cm), Male** | | | | | | |
| --- | --- | --- | --- | --- | --- | --- | --- | --- | --- | --- |
| **Age** | **L** | **M** | **S** | **P3** | **P10** | **P25** | **P50** | **P75** | **P90** | **P97** |
| 0 | -3,33073 | 47,12301 | 0,05692 | 42,78694 | 44,03976 | 45,46872 | 47,12301 | 49,07422 | 51,43192 | 54,37484 |
| 1 | -3,89105 | 51,21810 | 0,05372 | 46,82047 | 48,08191 | 49,52920 | 51,21810 | 53,23223 | 55,70466 | 58,86471 |
| 2 | -4,40014 | 54,94131 | 0,05050 | 50,53659 | 51,79481 | 53,24320 | 54,94131 | 56,98022 | 59,50866 | 62,79283 |
| 3 | -4,73586 | 57,98294 | 0,04738 | 53,61673 | 54,86500 | 56,30080 | 57,98294 | 60,00146 | 62,50385 | 65,75513 |
| 4 | -4,93247 | 60,36118 | 0,04457 | 56,06221 | 57,29590 | 58,71044 | 60,36118 | 62,33216 | 64,75935 | 67,88313 |
| 5 | -5,10440 | 62,23909 | 0,04219 | 58,02175 | 59,23627 | 60,62471 | 62,23909 | 64,15768 | 66,50571 | 69,50111 |
| 6 | -5,33185 | 63,82050 | 0,04025 | 59,68533 | 60,87794 | 62,23961 | 63,82050 | 65,69597 | 67,98621 | 70,89960 |
| 7 | -5,63835 | 65,21297 | 0,03871 | 61,15542 | 62,32476 | 63,66062 | 65,21297 | 67,05716 | 69,31426 | 72,19616 |
| 8 | -6,01601 | 66,47762 | 0,03743 | 62,49321 | 63,63864 | 64,94981 | 66,47762 | 68,29969 | 70,54247 | 73,43188 |
| 9 | -6,45224 | 67,65976 | 0,03633 | 63,74570 | 64,86687 | 66,15401 | 67,65976 | 69,46550 | 71,70642 | 74,63099 |
| 10 | -6,93419 | 68,78722 | 0,03534 | 64,94194 | 66,03875 | 67,30223 | 68,78722 | 70,57986 | 72,82647 | 75,80534 |
| 11 | -7,44270 | 69,85310 | 0,03444 | 66,07495 | 67,14777 | 68,38811 | 69,85310 | 71,63413 | 73,89022 | 76,93517 |
| 12 | -7,95346 | 70,83256 | 0,03361 | 67,11868 | 68,16862 | 69,38677 | 70,83256 | 72,60258 | 74,86907 | 77,98502 |
| 13 | -8,44800 | 71,71154 | 0,03287 | 68,05798 | 69,08665 | 70,28400 | 71,71154 | 73,47079 | 75,74701 | 78,93404 |
| 14 | -8,93244 | 72,52277 | 0,03219 | 68,92651 | 69,93518 | 71,11280 | 72,52277 | 74,27134 | 76,55656 | 79,81513 |
| 15 | -9,42869 | 73,31773 | 0,03152 | 69,77808 | 70,76716 | 71,92529 | 73,31773 | 75,05535 | 77,34943 | 80,68343 |
| 16 | -9,96434 | 74,14825 | 0,03084 | 70,66719 | 71,63615 | 72,77414 | 74,14825 | 75,87417 | 78,17751 | 81,59560 |
| 17 | -10,56563 | 75,05909 | 0,03011 | 71,64072 | 72,58833 | 73,70480 | 75,05909 | 76,77209 | 79,08544 | 82,60121 |
| 18 | -11,21870 | 76,02399 | 0,02936 | 72,67033 | 73,59612 | 74,69038 | 76,02399 | 77,72314 | 80,04691 | 83,67297 |
| 19 | -11,90102 | 77,00539 | 0,02862 | 73,71615 | 74,62042 | 75,69263 | 77,00539 | 78,69019 | 81,02404 | 84,76986 |
| 20 | -12,60419 | 77,99577 | 0,02788 | 74,77010 | 75,65340 | 76,70390 | 77,99577 | 79,66551 | 82,00827 | 85,88065 |
| 21 | -13,32703 | 79,00044 | 0,02715 | 75,83771 | 76,70057 | 77,72963 | 79,00044 | 80,65414 | 83,00365 | 87,00694 |
| 22 | -14,07066 | 80,02895 | 0,02642 | 76,92920 | 77,77197 | 78,77967 | 80,02895 | 81,66501 | 84,01767 | 88,15164 |
| 23 | -14,85437 | 81,12549 | 0,02564 | 78,09038 | 78,91297 | 79,89886 | 81,12549 | 82,74152 | 85,09244 | 89,35442 |
| 24 | -15,66949 | 82,28221 | 0,02485 | 79,31276 | 80,11530 | 81,07915 | 82,28221 | 83,87578 | 86,21899 | 90,59877 |

**Supplementary Table 12.** LMS parameters and head circumference percentiles (3rd, 10th, 25th, 50th, 75th, 90th, and 97th) for females with 22q11.2DS from birth to 24 months.

|  | | | | **Percentiles (HC, cm), Female** | | | | | | |
| --- | --- | --- | --- | --- | --- | --- | --- | --- | --- | --- |
| **Age** | **L** | **M** | **S** | **P3** | **P10** | **P25** | **P50** | **P75** | **P90** | **P97** |
| 0 | 6,75694 | 33,33698 | 0,03951 | 29,77565 | 31,23548 | 32,38396 | 33,33698 | 34,15496 | 34,87364 | 35,51599 |
| 1 | 5,43357 | 34,91827 | 0,04033 | 31,40199 | 32,76674 | 33,91799 | 34,91827 | 35,80562 | 36,60499 | 37,33372 |
| 2 | 4,14002 | 36,35637 | 0,04105 | 32,88600 | 34,16857 | 35,31563 | 36,35637 | 37,31117 | 38,19489 | 39,01870 |
| 3 | 2,90032 | 37,59878 | 0,04166 | 34,17794 | 35,38873 | 36,52552 | 37,59878 | 38,61678 | 39,58619 | 40,51247 |
| 4 | 1,74232 | 38,66234 | 0,04220 | 35,28934 | 36,43937 | 37,56306 | 38,66234 | 39,73889 | 40,79421 | 41,82964 |
| 5 | 0,69082 | 39,56565 | 0,04264 | 36,23663 | 37,33624 | 38,44595 | 39,56565 | 40,69524 | 41,83461 | 42,98365 |
| 6 | -0,23837 | 40,31801 | 0,04297 | 37,02956 | 38,08749 | 39,18310 | 40,31801 | 41,49392 | 42,71259 | 43,97589 |
| 7 | -1,03619 | 40,95095 | 0,04320 | 37,69848 | 38,72263 | 39,80507 | 40,95095 | 42,16607 | 43,45694 | 44,83091 |
| 8 | -1,69899 | 41,50358 | 0,04330 | 38,28191 | 39,27933 | 40,35014 | 41,50358 | 42,75056 | 44,10410 | 45,57990 |
| 9 | -2,21936 | 42,00839 | 0,04327 | 38,81092 | 39,78845 | 40,84996 | 42,00839 | 43,27971 | 44,68385 | 46,24606 |
| 10 | -2,59820 | 42,47961 | 0,04313 | 39,29865 | 40,26276 | 41,31780 | 42,47961 | 43,76828 | 45,20970 | 46,83800 |
| 11 | -2,84214 | 42,92110 | 0,04291 | 39,74815 | 40,70489 | 41,75663 | 42,92110 | 44,22113 | 45,68678 | 47,35860 |
| 12 | -2,95766 | 43,33376 | 0,04265 | 40,15963 | 41,11477 | 42,16663 | 43,33376 | 44,64020 | 46,11787 | 47,81028 |
| 13 | -2,94492 | 43,72006 | 0,04237 | 40,53456 | 41,49398 | 42,54973 | 43,72006 | 45,02864 | 46,50674 | 48,19688 |
| 14 | -2,80668 | 44,07452 | 0,04206 | 40,86837 | 41,83765 | 42,90070 | 44,07452 | 45,38081 | 46,84792 | 48,51371 |
| 15 | -2,54702 | 44,37894 | 0,04174 | 41,14389 | 42,12832 | 43,20174 | 44,37894 | 45,67843 | 47,12382 | 48,74572 |
| 16 | -2,17215 | 44,61734 | 0,04141 | 41,34562 | 42,35044 | 43,43705 | 44,61734 | 45,90575 | 47,32012 | 48,88267 |
| 17 | -1,68865 | 44,77824 | 0,04106 | 41,46338 | 42,49364 | 43,59575 | 44,77824 | 46,05110 | 47,42616 | 48,91749 |
| 18 | -1,10337 | 44,85672 | 0,04069 | 41,49329 | 42,55404 | 43,67348 | 44,85672 | 46,10947 | 47,43814 | 48,84999 |
| 19 | -0,42469 | 44,86308 | 0,04029 | 41,44532 | 42,54206 | 43,68061 | 44,86308 | 46,09168 | 47,36878 | 48,69689 |
| 20 | 0,33715 | 44,78786 | 0,03986 | 41,31091 | 42,44923 | 43,60816 | 44,78786 | 45,98853 | 47,21035 | 48,45349 |
| 21 | 1,16853 | 44,63255 | 0,03940 | 41,09096 | 42,27700 | 43,45747 | 44,63255 | 45,80244 | 46,96732 | 48,12735 |
| 22 | 2,05309 | 44,40864 | 0,03893 | 40,79611 | 42,03665 | 43,23977 | 44,40864 | 45,54597 | 46,65414 | 47,73527 |
| 23 | 2,96672 | 44,15131 | 0,03846 | 40,45993 | 41,76237 | 42,98948 | 44,15131 | 45,25593 | 46,30994 | 47,31876 |
| 24 | 3,88686 | 43,89161 | 0,03798 | 40,11328 | 41,48512 | 42,73724 | 43,89161 | 44,96444 | 45,96807 | 46,91216 |

**Supplementary Table 13.** LMS parameters and head circumference percentiles (3rd, 10th, 25th, 50th, 75th, 90th, and 97th) for males with 22q11.2DS from birth to 24 months.

|  | | | | **Percentiles (HC, cm), Male** | | | | | | |
| --- | --- | --- | --- | --- | --- | --- | --- | --- | --- | --- |
| **Age** | **L** | **M** | **S** | **P3** | **P10** | **P25** | **P50** | **P75** | **P90** | **P97** |
| 0 | 8,43626 | 35,20816 | 0,03551 | 31,59258 | 33,14333 | 34,29057 | 35,20816 | 35,97632 | 36,63898 | 37,22295 |
| 1 | 8,66510 | 36,77766 | 0,03340 | 33,28489 | 34,76546 | 35,87904 | 36,77766 | 37,53411 | 38,18913 | 38,76791 |
| 2 | 8,95218 | 38,27130 | 0,03132 | 34,91086 | 36,32132 | 37,39681 | 38,27130 | 39,01095 | 39,65350 | 40,22259 |
| 3 | 9,36311 | 39,62357 | 0,02930 | 36,39639 | 37,74287 | 38,77805 | 39,62357 | 40,34071 | 40,96485 | 41,51834 |
| 4 | 9,98041 | 40,77869 | 0,02734 | 37,67804 | 38,97161 | 39,96635 | 40,77869 | 41,46743 | 42,06660 | 42,59772 |
| 5 | 10,89038 | 41,72023 | 0,02547 | 38,73220 | 39,98786 | 40,94411 | 41,72023 | 42,37542 | 42,94353 | 43,44582 |
| 6 | 12,06806 | 42,50547 | 0,02370 | 39,61903 | 40,84733 | 41,76685 | 42,50547 | 43,12461 | 43,65869 | 44,12898 |
| 7 | 13,45938 | 43,18547 | 0,02203 | 40,39489 | 41,60088 | 42,48450 | 43,18547 | 43,76817 | 44,26779 | 44,70572 |
| 8 | 15,01367 | 43,77578 | 0,02048 | 41,07978 | 42,26427 | 43,11204 | 43,77578 | 44,32284 | 44,78905 | 45,19582 |
| 9 | 16,68250 | 44,30957 | 0,01906 | 41,70610 | 42,86878 | 43,68142 | 44,30957 | 44,82310 | 45,25823 | 45,63625 |
| 10 | 18,41808 | 44,80708 | 0,01778 | 42,29167 | 43,43256 | 44,21184 | 44,80708 | 45,29008 | 45,69723 | 46,04958 |
| 11 | 20,17438 | 45,27464 | 0,01665 | 42,84083 | 43,96058 | 44,70907 | 45,27464 | 45,73051 | 46,11306 | 46,44300 |
| 12 | 21,90853 | 45,70177 | 0,01567 | 43,34375 | 44,44255 | 45,16274 | 45,70177 | 46,13377 | 46,49486 | 46,80541 |
| 13 | 23,58530 | 46,08989 | 0,01482 | 43,80094 | 44,87954 | 45,57422 | 46,08989 | 46,50114 | 46,84373 | 47,13767 |
| 14 | 25,17973 | 46,45944 | 0,01409 | 44,23206 | 45,29184 | 45,96399 | 46,45944 | 46,85292 | 47,17980 | 47,45969 |
| 15 | 26,66950 | 46,79467 | 0,01347 | 44,62295 | 45,66469 | 46,31681 | 46,79467 | 47,17289 | 47,48637 | 47,75434 |
| 16 | 28,04186 | 47,09610 | 0,01293 | 44,97585 | 45,99955 | 46,63363 | 47,09610 | 47,46114 | 47,76315 | 48,02097 |
| 17 | 29,29915 | 47,38655 | 0,01246 | 45,31573 | 46,32031 | 46,93775 | 47,38655 | 47,74010 | 48,03222 | 48,28136 |
| 18 | 30,44838 | 47,69198 | 0,01204 | 45,66509 | 46,65190 | 47,25484 | 47,69198 | 48,03582 | 48,31963 | 48,56151 |
| 19 | 31,50058 | 48,01591 | 0,01169 | 46,02608 | 46,99774 | 47,58851 | 48,01591 | 48,35166 | 48,62857 | 48,86442 |
| 20 | 32,47269 | 48,31096 | 0,01137 | 46,35670 | 47,31305 | 47,89244 | 48,31096 | 48,63943 | 48,91017 | 49,14066 |
| 21 | 33,37670 | 48,49989 | 0,01107 | 46,58571 | 47,52341 | 48,09055 | 48,49989 | 48,82100 | 49,08559 | 49,31078 |
| 22 | 34,21994 | 48,60649 | 0,01080 | 46,73418 | 47,65165 | 48,20629 | 48,60649 | 48,92038 | 49,17896 | 49,39902 |
| 23 | 35,01534 | 48,66872 | 0,01055 | 46,83763 | 47,73481 | 48,27729 | 48,66872 | 48,97572 | 49,22861 | 49,44381 |
| 24 | 35,78569 | 48,71018 | 0,01031 | 46,92043 | 47,79687 | 48,32732 | 48,71018 | 49,01050 | 49,25790 | 49,46843 |

**Supplementary Table 14.** LMS parameters and weight percentiles (3rd, 10th, 25th, 50th, 75th, 90th, and 97th) for females with 22q11.2DS from 2 to 17 years of age.

|  | | | | **Percentiles (Weight, kg), Female** | | | | | | |
| --- | --- | --- | --- | --- | --- | --- | --- | --- | --- | --- |
| **Age** | **L** | **M** | **S** | **P3** | **P10** | **P25** | **P50** | **P75** | **P90** | **P97** |
| 2 | 0,67420 | 10,61436 | 0,16272 | 7,35063 | 8,39497 | 9,48349 | 10,61436 | 11,78593 | 12,99676 | 14,24552 |
| 3 | 0,47504 | 12,23653 | 0,17767 | 8,29127 | 9,51716 | 10,83206 | 12,23653 | 13,73114 | 15,31639 | 16,99278 |
| 4 | 0,28414 | 13,89464 | 0,19239 | 9,24421 | 10,64554 | 12,19275 | 13,89464 | 15,76018 | 17,79853 | 20,01900 |
| 5 | 0,11483 | 16,01219 | 0,20664 | 10,48482 | 12,10197 | 13,93613 | 16,01219 | 18,35748 | 21,00186 | 23,97801 |
| 6 | -0,01656 | 18,82508 | 0,21960 | 12,15288 | 14,05655 | 16,26414 | 18,82508 | 21,79701 | 25,24712 | 29,25381 |
| 7 | -0,09548 | 22,00833 | 0,22986 | 14,03393 | 16,26998 | 18,90227 | 22,00833 | 25,68231 | 30,03892 | 35,21828 |
| 8 | -0,10889 | 25,32231 | 0,23623 | 15,97403 | 18,57812 | 21,66135 | 25,32231 | 29,68206 | 34,88987 | 41,13036 |
| 9 | -0,05352 | 28,68128 | 0,23799 | 17,92510 | 20,93815 | 24,48954 | 28,68128 | 33,63578 | 39,50024 | 46,45190 |
| 10 | 0,05757 | 32,12761 | 0,23504 | 19,94825 | 23,41687 | 27,44835 | 32,12761 | 37,55144 | 43,83003 | 51,08866 |
| 11 | 0,20852 | 35,68650 | 0,22780 | 22,11000 | 26,07504 | 30,58292 | 35,68650 | 41,44184 | 47,90829 | 55,14855 |
| 12 | 0,38264 | 39,46764 | 0,21700 | 24,55357 | 29,04442 | 34,00981 | 39,46764 | 45,43544 | 51,93033 | 58,96912 |
| 13 | 0,57108 | 43,56764 | 0,20357 | 27,40972 | 32,43924 | 37,82781 | 43,56764 | 49,65175 | 56,07379 | 62,82796 |
| 14 | 0,76691 | 47,98635 | 0,18829 | 30,76852 | 36,30811 | 42,05256 | 47,98635 | 54,09657 | 60,37226 | 66,80399 |
| 15 | 0,96283 | 52,67356 | 0,17167 | 34,71799 | 40,67180 | 46,65829 | 52,67356 | 58,71452 | 64,77871 | 70,86411 |
| 16 | 1,15496 | 57,47964 | 0,15409 | 39,27114 | 45,46256 | 51,52554 | 57,47964 | 63,33948 | 69,11636 | 74,81926 |
| 17 | 1,34417 | 62,29972 | 0,13600 | 44,40508 | 50,60743 | 56,55783 | 62,29972 | 67,86472 | 73,27670 | 78,55434 |

**Supplementary Table 15.** LMS parameters and weight percentiles (3rd, 10th, 25th, 50th, 75th, 90th, and 97th) for males with 22q11.2DS from 2 to 17 years of age.

|  | | | | **Percentiles (Weight, kg), Male** | | | | | | |
| --- | --- | --- | --- | --- | --- | --- | --- | --- | --- | --- |
| **Age** | **L** | **M** | **S** | **P3** | **P10** | **P25** | **P50** | **P75** | **P90** | **P97** |
| 2 | -0,55052 | 11,82202 | 0,10214 | 9,74128 | 10,36711 | 11,05756 | 11,82202 | 12,67166 | 13,61987 | 14,68280 |
| 3 | -0,72267 | 13,82018 | 0,10996 | 11,26866 | 12,02240 | 12,86740 | 13,82018 | 14,90135 | 16,13693 | 17,56024 |
| 4 | -0,88909 | 15,79904 | 0,11817 | 12,74867 | 13,63294 | 14,64068 | 15,79904 | 17,14363 | 18,72206 | 20,59949 |
| 5 | -1,05966 | 17,99357 | 0,12727 | 14,36283 | 15,39364 | 16,58937 | 17,99357 | 19,66672 | 21,69534 | 24,20788 |
| 6 | -1,23954 | 20,30295 | 0,13740 | 16,02689 | 17,21300 | 18,61559 | 20,30295 | 22,37629 | 24,99271 | 28,41078 |
| 7 | -1,42738 | 22,55383 | 0,14795 | 17,62066 | 18,95551 | 20,56567 | 22,55383 | 25,08356 | 28,43408 | 33,12872 |
| 8 | -1,60841 | 24,80473 | 0,15803 | 19,21084 | 20,68833 | 22,50426 | 24,80473 | 27,84017 | 32,08422 | 38,57135 |
| 9 | -1,76613 | 27,22096 | 0,16668 | 20,94382 | 22,56728 | 24,59441 | 27,22096 | 30,80564 | 36,09737 | 45,02267 |
| 10 | -1,89987 | 30,13435 | 0,17335 | 23,08783 | 24,87954 | 27,14475 | 30,13435 | 34,33472 | 40,85610 | 53,06565 |
| 11 | -2,00421 | 33,63710 | 0,17803 | 25,70999 | 27,70041 | 30,23956 | 33,63710 | 38,51986 | 46,42629 | 62,77774 |
| 12 | -2,07193 | 37,57798 | 0,18084 | 28,68809 | 30,90279 | 33,74369 | 37,57798 | 43,16987 | 52,48837 | 73,28802 |
| 13 | -2,10607 | 41,69007 | 0,18203 | 31,81766 | 34,26817 | 37,41954 | 41,69007 | 47,96205 | 58,56361 | 83,21698 |
| 14 | -2,11274 | 45,78504 | 0,18196 | 34,95261 | 37,64053 | 41,09796 | 45,78504 | 52,67379 | 64,33636 | 91,59240 |
| 15 | -2,09944 | 49,77078 | 0,18099 | 38,02205 | 40,94342 | 44,69563 | 49,77078 | 57,20105 | 69,68346 | 98,21275 |
| 16 | -2,07321 | 53,65320 | 0,17942 | 41,02888 | 44,17945 | 48,21568 | 53,65320 | 61,56009 | 74,66563 | 103,51670 |
| 17 | -2,04061 | 57,48831 | 0,17749 | 44,01611 | 47,39349 | 51,70649 | 57,48831 | 65,82646 | 79,42616 | 108,11650 |

**Supplementary Table 16.** LMS parameters and height percentiles (3rd, 10th, 25th, 50th, 75th, 90th, and 97th) for females with 22q11.2DS from 2 to 17 years of age.

|  | | | | **Percentiles (Height, cm), Female** | | | | | | |
| --- | --- | --- | --- | --- | --- | --- | --- | --- | --- | --- |
| **Age** | **L** | **M** | **S** | **P3** | **P10** | **P25** | **P50** | **P75** | **P90** | **P97** |
| 2 | 3,8805 | 86,0565 | 0,0475 | 76,4361 | 80,0219 | 83,1967 | 86,0565 | 88,6662 | 91,0716 | 93,3068 |
| 3 | 2,5614 | 91,4394 | 0,0474 | 82,0289 | 85,3470 | 88,4751 | 91,4394 | 94,2607 | 96,9561 | 99,5392 |
| 4 | 1,3516 | 97,0151 | 0,0475 | 87,6357 | 90,7998 | 93,9255 | 97,0151 | 100,0704 | 103,0933 | 106,0853 |
| 5 | 0,3046 | 103,7828 | 0,0480 | 94,1559 | 97,2926 | 100,5013 | 103,7828 | 107,1381 | 110,5681 | 114,0738 |
| 6 | -0,3890 | 111,7775 | 0,0487 | 101,5822 | 104,8316 | 108,2272 | 111,7775 | 115,4917 | 119,3794 | 123,4514 |
| 7 | -0,5633 | 118,8681 | 0,0495 | 107,9550 | 111,4123 | 115,0459 | 118,8681 | 122,8927 | 127,1344 | 131,6096 |
| 8 | -0,3398 | 124,6431 | 0,0501 | 112,9564 | 116,6823 | 120,5747 | 124,6431 | 128,8976 | 133,3489 | 138,0085 |
| 9 | 0,1764 | 130,1893 | 0,0503 | 117,6278 | 121,6988 | 125,8851 | 130,1893 | 134,6140 | 139,1617 | 143,8353 |
| 10 | 0,9882 | 136,1285 | 0,0501 | 122,4943 | 127,0372 | 131,5819 | 136,1285 | 140,6769 | 145,2271 | 149,7788 |
| 11 | 2,0070 | 141,8390 | 0,0496 | 126,9893 | 132,1261 | 137,0692 | 141,8390 | 146,4525 | 150,9241 | 155,2661 |
| 12 | 3,0820 | 146,8979 | 0,0489 | 130,7634 | 136,5887 | 141,9381 | 146,8979 | 151,5315 | 155,8876 | 160,0040 |
| 13 | 4,0885 | 151,1071 | 0,0482 | 133,6947 | 140,2605 | 145,9946 | 151,1071 | 155,7351 | 159,9735 | 163,8910 |
| 14 | 4,9442 | 154,3308 | 0,0475 | 135,7323 | 143,0317 | 149,1025 | 154,3308 | 158,9415 | 163,0780 | 166,8378 |
| 15 | 5,6440 | 156,8023 | 0,0470 | 137,1260 | 145,1254 | 151,4846 | 156,8023 | 161,3945 | 165,4496 | 169,0897 |
| 16 | 6,2175 | 158,7611 | 0,0466 | 138,1038 | 146,7654 | 153,3723 | 158,7611 | 163,3366 | 167,3273 | 170,8757 |
| 17 | 6,7137 | 160,4285 | 0,0463 | 138,8302 | 148,1475 | 154,9789 | 160,4285 | 164,9892 | 168,9263 | 172,3999 |

**Supplementary Table 17.** LMS parameters and height percentiles (3rd, 10th, 25th, 50th, 75th, 90th, and 97th) for males with 22q11.2DS from 2 to 17 years of age.

|  | | | | **Percentiles (Height, cm), Male** | | | | | | |
| --- | --- | --- | --- | --- | --- | --- | --- | --- | --- | --- |
| **Age** | **L** | **M** | **S** | **P3** | **P10** | **P25** | **P50** | **P75** | **P90** | **P97** |
| 2 | -4,7566 | 86,2838 | 0,0550 | 78,9754 | 81,0211 | 83,4151 | 86,2838 | 89,8331 | 94,4314 | 100,8329 |
| 3 | -3,9360 | 93,2259 | 0,0564 | 84,9203 | 87,2849 | 90,0148 | 93,2259 | 97,0958 | 101,9159 | 108,2091 |
| 4 | -3,1951 | 99,9757 | 0,0577 | 90,6235 | 93,3312 | 96,4144 | 99,9757 | 104,1632 | 109,2015 | 115,4512 |
| 5 | -2,6023 | 106,4710 | 0,0589 | 96,0731 | 99,1275 | 102,5634 | 106,4710 | 110,9744 | 116,2494 | 122,5562 |
| 6 | -2,1742 | 112,4887 | 0,0600 | 101,1190 | 104,4959 | 108,2589 | 112,4887 | 117,2920 | 122,8134 | 129,2543 |
| 7 | -1,8511 | 117,8477 | 0,0607 | 105,6291 | 109,2910 | 113,3397 | 117,8477 | 122,9076 | 128,6406 | 135,2083 |
| 8 | -1,5538 | 122,7969 | 0,0609 | 109,8371 | 113,7578 | 118,0569 | 122,7969 | 128,0553 | 133,9299 | 140,5456 |
| 9 | -1,1766 | 127,6875 | 0,0604 | 114,0428 | 118,2263 | 122,7588 | 127,6875 | 133,0682 | 138,9682 | 145,4691 |
| 10 | -0,6172 | 132,8445 | 0,0591 | 118,5207 | 123,0059 | 127,7722 | 132,8445 | 138,2506 | 144,0216 | 150,1924 |
| 11 | 0,0899 | 138,4085 | 0,0568 | 123,4748 | 128,2812 | 133,2573 | 138,4085 | 143,7403 | 149,2583 | 154,9685 |
| 12 | 0,8853 | 144,1250 | 0,0535 | 128,8047 | 133,8895 | 138,9964 | 144,1250 | 149,2746 | 154,4447 | 159,6347 |
| 13 | 1,6983 | 149,5131 | 0,0493 | 134,2104 | 139,4412 | 144,5384 | 149,5131 | 154,3748 | 159,1318 | 163,7915 |
| 14 | 2,4921 | 154,2629 | 0,0447 | 139,4293 | 144,6275 | 149,5611 | 154,2629 | 158,7602 | 163,0750 | 167,2258 |
| 15 | 3,2553 | 158,3370 | 0,0399 | 144,3693 | 149,3568 | 153,9945 | 158,3370 | 162,4264 | 166,2959 | 169,9724 |
| 16 | 3,9929 | 161,9249 | 0,0351 | 149,1047 | 153,7399 | 157,9910 | 161,9249 | 165,5920 | 169,0309 | 172,2724 |
| 17 | 4,7128 | 165,2653 | 0,0305 | 153,7757 | 157,9575 | 161,7645 | 165,2653 | 168,5107 | 171,5393 | 174,3814 |

**Supplementary Table 18.** LMS parameters and BMI percentiles (3rd, 10th, 25th, 50th, 75th, 90th, and 97th) for females with 22q11.2DS from 2 to 17 years of age.

|  | | | | **Percentiles (BMI, kg/m²), Female** | | | | | | |
| --- | --- | --- | --- | --- | --- | --- | --- | --- | --- | --- |
| **Age** | **L** | **M** | **S** | **P3** | **P10** | **P25** | **P50** | **P75** | **P90** | **P97** |
| 2 | -0,65273 | 15,26621 | 0,07843 | 13,14840 | 13,79741 | 14,50113 | 15,26621 | 16,10038 | 17,01266 | 18,01365 |
| 3 | -0,56744 | 15,21188 | 0,08345 | 12,96959 | 13,65597 | 14,40105 | 15,21188 | 16,09662 | 17,06476 | 18,12740 |
| 4 | -0,44968 | 15,21590 | 0,08853 | 12,83230 | 13,56265 | 14,35483 | 15,21590 | 16,15391 | 17,17813 | 18,29925 |
| 5 | -0,28121 | 15,35166 | 0,09369 | 12,78914 | 13,57780 | 14,42986 | 15,35166 | 16,35028 | 17,43367 | 18,61077 |
| 6 | -0,05913 | 15,64664 | 0,09889 | 12,85356 | 13,72079 | 14,65024 | 15,64664 | 16,71510 | 17,86114 | 19,09075 |
| 7 | 0,20094 | 16,03214 | 0,10385 | 12,96724 | 13,93159 | 14,95241 | 16,03214 | 17,17328 | 18,37842 | 19,65020 |
| 8 | 0,45948 | 16,44496 | 0,10834 | 13,08893 | 14,16170 | 15,28028 | 16,44496 | 17,65601 | 18,91371 | 20,21831 |
| 9 | 0,68312 | 16,83802 | 0,11205 | 13,20230 | 14,38296 | 15,59517 | 16,83802 | 18,11065 | 19,41227 | 20,74216 |
| 10 | 0,82523 | 17,30654 | 0,11502 | 13,40955 | 14,68910 | 15,98845 | 17,30654 | 18,64243 | 19,99526 | 21,36430 |
| 11 | 0,85901 | 17,88713 | 0,11753 | 13,75655 | 15,11628 | 16,49349 | 17,88713 | 19,29626 | 20,72006 | 22,15779 |
| 12 | 0,78916 | 18,53256 | 0,11979 | 14,21028 | 15,62397 | 17,06519 | 18,53256 | 20,02486 | 21,54099 | 23,07998 |
| 13 | 0,64789 | 19,18639 | 0,12203 | 14,71003 | 16,15550 | 17,64803 | 19,18639 | 20,76948 | 22,39624 | 24,06573 |
| 14 | 0,47025 | 19,84275 | 0,12436 | 15,23069 | 16,69640 | 18,23362 | 19,84275 | 21,52414 | 23,27814 | 25,10508 |
| 15 | 0,27593 | 20,51612 | 0,12685 | 15,77121 | 17,25322 | 18,83355 | 20,51612 | 22,30495 | 24,20407 | 26,21762 |
| 16 | 0,07662 | 21,19427 | 0,12942 | 16,31802 | 17,81440 | 19,43660 | 21,19427 | 23,09771 | 25,15792 | 27,38668 |
| 17 | -0,12275 | 21,87284 | 0,13205 | 16,86631 | 18,37595 | 20,03897 | 21,87284 | 23,89728 | 26,13449 | 28,60957 |

**Supplementary Table 19.** LMS parameters and BMI percentiles (3rd, 10th, 25th, 50th, 75th, 90th, and 97th) for males with 22q11.2DS from 2 to 17 years of age.

|  | | | | **Percentiles (BMI, kg/m²), Male** | | | | | | |
| --- | --- | --- | --- | --- | --- | --- | --- | --- | --- | --- |
| **Age** | **L** | **M** | **S** | **P3** | **P10** | **P25** | **P50** | **P75** | **P90** | **P97** |
| 2 | 2,74213 | 15,81589 | 0,07761 | 12,92027 | 14,00347 | 14,95752 | 15,81589 | 16,59995 | 17,32431 | 17,99941 |
| 3 | 2,27469 | 15,75862 | 0,07960 | 12,93225 | 13,95610 | 14,89215 | 15,75862 | 16,56824 | 17,33033 | 18,05192 |
| 4 | 1,80726 | 15,71894 | 0,08175 | 12,94936 | 13,92188 | 14,84239 | 15,71894 | 16,55769 | 17,36346 | 18,14010 |
| 5 | 1,33982 | 15,77853 | 0,08428 | 13,03441 | 13,96922 | 14,88323 | 15,77853 | 16,65688 | 17,51976 | 18,36842 |
| 6 | 0,87239 | 15,97764 | 0,08742 | 13,21663 | 14,12949 | 15,04994 | 15,97764 | 16,91227 | 17,85353 | 18,80118 |
| 7 | 0,40495 | 16,27177 | 0,09098 | 13,46922 | 14,36847 | 15,30252 | 16,27177 | 17,27665 | 18,31756 | 19,39491 |
| 8 | -0,06249 | 16,57499 | 0,09441 | 13,73805 | 14,62160 | 15,56578 | 16,57499 | 17,65401 | 18,80796 | 20,04239 |
| 9 | -0,52992 | 16,87035 | 0,09695 | 14,02678 | 14,88739 | 15,83148 | 16,87035 | 18,01726 | 19,28791 | 20,70097 |
| 10 | -0,99736 | 17,24172 | 0,09816 | 14,41156 | 15,24587 | 16,18257 | 17,24172 | 18,44900 | 19,83781 | 21,45238 |
| 11 | -1,46480 | 17,67119 | 0,09805 | 14,87286 | 15,67881 | 16,60172 | 17,67119 | 18,92823 | 20,43138 | 22,26764 |
| 12 | -1,93223 | 18,07020 | 0,09672 | 15,33140 | 16,10467 | 17,00471 | 18,07020 | 19,35858 | 20,95967 | 23,02357 |
| 13 | -2,39967 | 18,36175 | 0,09427 | 15,71661 | 16,45134 | 17,31768 | 18,36175 | 19,65635 | 21,32521 | 23,60092 |
| 14 | -2,86711 | 18,61290 | 0,09092 | 16,07881 | 16,77366 | 17,60120 | 18,61290 | 19,89443 | 21,60247 | 24,06711 |
| 15 | -3,33454 | 18,88197 | 0,08687 | 16,46350 | 17,12022 | 17,90809 | 18,88197 | 20,13705 | 21,85891 | 24,48170 |
| 16 | -3,80198 | 19,15575 | 0,08238 | 16,85534 | 17,47568 | 18,22369 | 19,15575 | 20,37300 | 22,08305 | 24,81899 |
| 17 | -4,26942 | 19,41090 | 0,07774 | 17,22892 | 17,81452 | 18,52304 | 19,41090 | 20,58190 | 22,25811 | 25,05707 |
